# Sensitive and reproducible MEG resting-state metrics of functional connectivity in Alzheimer’s disease

**DOI:** 10.1186/s13195-022-00970-4

**Published:** 2022-02-26

**Authors:** Deborah N. Schoonhoven, Casper T. Briels, Arjan Hillebrand, Philip Scheltens, Cornelis J. Stam, Alida A. Gouw

**Affiliations:** 1 Alzheimer Center Amsterdam, Department of Neurology, Amsterdam Neuroscience, Vrije Universiteit Amsterdam, Amsterdam UMC, Amsterdam, The Netherlands; 2 Department of Clinical Neurophysiology and MEG Center, Department of Neurology, Amsterdam Neuroscience, Vrije Universiteit Amsterdam, Amsterdam UMC, Amsterdam, The Netherlands

**Keywords:** Alzheimer’s disease, Subjective cognitive decline, Magnetoencephalography, Functional connectivity, Reproducibility, Sensitivity

## Abstract

**Background:**

Analysis of functional brain networks in Alzheimer’s disease (AD) has been hampered by a lack of reproducible, yet valid metrics of functional connectivity (FC). This study aimed to assess both the sensitivity and reproducibility of the corrected amplitude envelope correlation (AEC-c) and phase lag index (PLI), two metrics of FC that are insensitive to the effects of volume conduction and field spread, in two separate cohorts of patients with dementia due to AD versus healthy elderly controls.

**Methods:**

Subjects with a clinical diagnosis of AD dementia with biomarker proof, and a control group of subjective cognitive decline (SCD), underwent two 5-min resting-state MEG recordings. Data consisted of a test (AD = 28; SCD = 29) and validation (AD = 29; SCD = 27) cohort. Time-series were estimated for 90 regions of interest (ROIs) in the automated anatomical labelling (AAL) atlas. For each of five canonical frequency bands, the AEC-c and PLI were calculated between all 90 ROIs, and connections were averaged per ROI. General linear models were constructed to compare the global FC differences between the groups, assess the reproducibility, and evaluate the effects of age and relative power. Reproducibility of the regional FC differences was assessed using the Mann-Whitney *U* tests, with correction for multiple testing using the false discovery rate (FDR).

**Results:**

The AEC-c showed significantly and reproducibly lower global FC for the AD group compared to SCD, in the alpha (8–13 Hz) and beta (13–30 Hz) bands, while the PLI revealed reproducibly lower FC for the AD group in the delta (0.5–4 Hz) band and higher FC for the theta (4–8 Hz) band. Regionally, the beta band AEC-c showed reproducibility for almost all ROIs (except for 13 ROIs in the frontal and temporal lobes). For the other bands, the AEC-c and PLI did not show regional reproducibility after FDR correction. The theta band PLI was susceptible to the effect of relative power.

**Conclusion:**

For MEG, the AEC-c is a sensitive and reproducible metric, able to distinguish FC differences between patients with AD dementia and cognitively healthy controls. These two measures likely reflect different aspects of neural activity and show differential sensitivity to changes in neural dynamics.

**Supplementary Information:**

The online version contains supplementary material available at 10.1186/s13195-022-00970-4.

## Introduction

Cognitive functioning requires coordinated interaction between neurons within and across specialized brain areas [1]. In order to achieve this coordination, neuronal populations need to be functionally connected, which is defined as a statistical dependency between (remote) neurophysiological signals being present [2]. The signals recorded from neuronal populations often consist of oscillations that cover a broad frequency spectrum. In order to assess the connectivity within and between the oscillatory systems, a degree of synchronization must be present (often measured through amplitude or phase correlation). Phase or amplitude correlation allows for effective neuronal coordination and therefore normal cognitive functioning [3–5].

Alzheimer’s disease (AD), the most common form of dementia, is characterized by pathologically accumulating amyloid-β and hyperphosphorylated tau in the brain, leading to synaptic dysfunction [6–8]. Synaptic perturbation as present in AD patients is thought to disturb neuronal synchronization. As a result, communication within and between the brain areas is disrupted, leading to cognitive impairment. In previous studies, resting-state functional networks have indeed been shown to be altered in AD patients [9–12]. In order to better understand the disease process—and find novel therapeutic targets—it is of importance to gain an improved understanding of the role of disrupted synaptic function in AD.

Both electroencephalography (EEG) and magnetoencephalography (MEG) are attractive modalities to address these challenges; both techniques carry low-risk as well as low-burden to patients and are non-invasive. Both directly measure neuronal activity with millisecond temporal resolution, allowing for the reconstruction of oscillatory neuronal activity, and functional brain networks [13, 14]. An added benefit of MEG is the lack of need for a reference, aiding interpretability. Additionally, while skull and scalp tissue perturb EEG potentials, they do not affect magnetic fields [13]. Finally, recent studies have demonstrated that MEG can detect signals originating from the deeper brain regions by projecting data into ‘source space’ [15–20], thereby offering insight into neuronal functioning at both the cortical and subcortical levels.

While M/EEG-derived metrics show potential as markers of neurodegeneration, such markers are only useful if they have a high reproducibility (in the context of this paper defined as reproducing a given pattern of AD versus elderly healthy control functional connectivity (FC) differences) and sensitivity (discriminatory value between AD and elderly healthy controls). Previous studies have already established the connection between reduced cognitive performance and (sub)cortical oscillatory slowing in MEG and EEG [17, 21, 22], reflected by higher relative power in the lower (delta and theta) frequency bands, lower relative power in the higher (alpha and beta) frequency bands, and lower peak frequency. However, when it comes to the analysis of FC or networks in AD, the lack of reproducible metrics of functional connectivity has led to conflicting results [23, 24]. Nonetheless, the importance of reproducible estimates of FC in M/EEG literature is increasingly recognized [25], with several studies having provided significant insights. Marquetand and co-workers assessed the reliability (defined as test-retest reliability, as well as methodological and technical influencing factors) of the imaginary part of coherency and the weighted phase-lag index and found a strong increase of reliability with more trials (10-s data segments). For MEG, global reliability for both metrics was excellent in the alpha band, as well as high-density EEG, with the delta band being the worst. Finally, they found lower reliability for both metrics in a vertex-based regional analysis, compared to global reliability [26]. Additionally, Colclough and colleagues tested 12 metrics of MEG functional connectivity against the criteria of individual- and group-level reproducibility again using a test-retest design [27]. Overall, phase-based metrics and imaginary partial coherence performed worst, while the leakage-corrected amplitude envelope correlation (AEC-c) showed reproducible results. Recently, we evaluated the reproducibility of disease-associated effects for several functional connectivity metrics in two EEG cohorts of AD dementia patients and subjective cognitive decline (SCD) subjects [24]. The AEC-c (in the alpha and beta band) was the most reproducible metric, and it also correlated with disease severity and was not influenced by relative band power, demographic variables, (co)morbidities, or interfering medication. Important advantage of the current study is MEG’s high spatial resolution compared to EEG and the opportunity to investigate reproducibility on regional cortical and subcortical levels.

In this study, we evaluated two metrics of FC, in source space, in two clinically derived MEG cohorts of AD dementia and elderly control subjects, using the AEC-c and the phase lag index (PLI). These metrics were selected based on their performance in previous work [24, 27, 28], and their use in AD literature [11, 29–35]. These two measures likely reflect different aspects of neural activity and show differential sensitivity to changes in neural dynamics [36]. Power envelope correlations have shown a good correlation to BOLD signals, which, in turn, have been matched with known structural connectivity [5]. Phase-based measures seem less determined by structural connectivity and more related to stimulus context, task, or cognitive setting. Spatially, an MEG study using the phase lag index revealed patterns of highly connected regions that differed across frequency bands [37]. In the alpha band, the most strongly connected regions were the visual and posterior cingulate cortex. In the beta band, this involved the sensorimotor and parietal cortex, and in the gamma band, the temporal and parietal areas showed high functional connectivity. In contrast, analysis of orthogonalized power envelope correlations showed prominent hubs in the dorsolateral prefrontal, lateral parietal, and temporal cortex for the beta band, while theta band interactions involved major hubs in the medial temporal lobe and gamma band hubs in the sensorimotor cortex [38]. Both metrics are insensitive to the effects of volume conduction and field spread. We chose only to use metrics that are immune to these effects, because there is increasing evidence that interpretable connectivity estimation is only possible when zero-lag connections are removed or ignored [27]. Signal leakage has a profound impact on FC estimates, resulting in spurious connections that in turn causes inflated measures of consistency. Interpretation of FC estimates is therefore problematic when metrics are used that do not address this problem. We first assessed sensitivity, i.e. which metric could identify significant group FC differences and patterns in a test cohort with sufficiently large effect size, both on a global and regional levels. If the effect size is adequately large in a cohort with relatively large contrasts (AD versus SCD), it may also be suitable to capture smaller contrasts (i.e. MCI versus SCD) in future studies. Secondly, we repeated the analysis in a second independent cohort to see if these effects would reproduce. Thirdly, we assessed whether covariates age and relative power significantly influenced the observed group differences. Finally, to see which metric correlated with global cognitive performance, correlations with Mini-Mental State Examination (MMSE) scores were calculated. Based on previous findings, we expected that the AEC-c would provide the most robust results, mainly for the alpha and beta bands.

## Methods

### Participants

Subjects were recruited from the Amsterdam Dementia Cohort (ADC), consisting of patients who visited the memory clinic in the Alzheimer Centre VUmc, Amsterdam UMC, between May 2015 and March 2018. Subjects followed a standard dementia screening protocol which consisted of history taking, neurological examination, blood tests, neuropsychological tests, magnetic resonance imaging (MRI), EEG or MEG recording, and, if possible, lumbar punction to obtain cerebrospinal fluid (CSF) and/or positron emission tomography (PET) [39]. The local Medical Ethics Committee has approved a general protocol for biobanking and use of the clinical data for research purposes. All subjects gave written informed consent for the use of their data for research purposes.

Subjects were included if they had received a clinical diagnosis of probable Alzheimer’s Dementia (AD) or SCD during a multidisciplinary meeting consisting of a neurologist, radiologist, neuropsychologist, clinical neurophysiologist, nurse, and psychiatrist [40]. SCD subjects were included as elderly controls. The presence of Alzheimer’s disease pathology was subsequently verified using CSF and/or amyloid-PET, if available. In order to classify as amyloid-positive, subjects had to have either positive CSF Aβ 1–42 and/or positive amyloid-PET. For CSF Aβ 1–42, drift-corrected values were used; the cut-off was set at 813 pg/ml [41]. When both amyloid-PET and CSF data were available, amyloid-PET was decisive. For positive tau pathology, p-tau values were used; the cut-off was set at 52 pg/ml. Potential AD subjects were excluded if they were amyloid- and tau-negative. Potential SCD subjects were excluded if they were positive for amyloid and tau pathology according to the aforementioned criteria. In case there was no biomarker information available, follow-up visits were examined (if available) to see if the diagnosis had remained unchanged. For the purpose of internal validation, subjects were randomly allocated to either of the two cohorts, thus creating a test and validation cohort of approximately equal size. Although the allocation of subjects was random, care was taken to select a sample where there was as little difference as possible between the diagnostic groups, in order to retain equal conditions for reproducibility.

### Data acquisition

#### MEG recordings

MEG data were acquired with a 306-channel whole-head MEG system (Elekta Neuromag Oy, Helsinki, Finland), while subjects were in the supine position in a magnetically shielded room (VacuumSchmelze GmbH, Hanua, Germany). For each subject, two 5-min mainly eyes-closed resting-state recordings were made. Subjects were instructed to open and close their eyes several times on cue during the recordings for clinical purposes (i.e. to assess the reactivity of the alpha rhythm). Magnetic fields were recorded at a sample frequency of 1250 Hz, with an anti-aliasing filter of 410 Hz and a high-pass filter of 0.1 Hz. The subjects’ head position in relation to the MEG sensors was recorded using signals from four or five head localization coils.

#### Pre-processing of MEG data

Raw MEG data were visually inspected for malfunctioning and noisy channels, which were subsequently removed, after which, the temporal extension of Signal Space Separation (tSSS) in the MaxFilter software (Elekta Neuromag Oy, version 2.2.15) [42] was applied, as well as a broad-band filter (0.5–100 Hz).

Subjects’ MEG data were co-registered with a best-matching template MRI using surface matching, with an estimated resulting accuracy of 4 mm [43]. The MRI templates were custom-built using 3D T1-weighted MRI images and sized extra small, small, medium, and large. A single sphere was fitted to the outline of the scalp as obtained from the co-registered MRI, which was used as a volume conductor model for the atlas-based beamformer approach [37] that was used in order to reconstruct neuronal activity in cortical and subcortical regions. Based on the automatic anatomical labelling (AAL) atlas [44, 45], 78 cortical and 12 deep grey matter regions of interest (ROIs) were defined in a subject’s co-registered MRI, using the centroid voxel for each ROI [46]. For each of these centroid voxels, time-series (i.e. virtual electrodes) of neuronal activity were reconstructed by projecting sensor signals to source space using beamforming [47, 48]. The broadband data were used for the estimation of the beamformer weights, in order to avoid overestimation of covariance between channels [49] as well as a unity noise covariance matrix, and an equivalent current dipole as source model. On average, 302 s of data (range 270–343 s) were used for the estimation of the data covariance matrix, which was regularized using singular value truncation with the default setting of 1e−06. The optimum orientation of the equivalent current dipole was found using singular value decomposition [50]. The broadband sensor-level data were subsequently projected through the normalized beamformer weights [51] resulting in a time series of neuronal activity for each ROI.

#### Time-series analyses

The time series for these ROIs were downsampled by a factor of 4 and used for further analysis. For each subject, 10 non-overlapping, artefact-free, eyes-closed epochs of 4096 samples (13.1072 s) were selected, based on careful visual inspection by an experienced assessor (AG). First, all epochs received a quality score of 1 to 4: 1 = no eye movement, muscle artefacts, signs of drowsiness, or other artefacts; 2 = minimal presence of artefacts; 3 = moderate presence of artefacts; and 4 = strong presence of artefacts. For a more detailed description of the selection method, see also [52]. A second assessor (DS) subsequently selected ten epochs with the highest quality score for each subject. The optimal epoch length was based on previous work, which established a minimum length of 6 s for the AEC and 10 s for the PLI as the onset of stability for estimates of functional connectivity at the source level [53]. Inspection and further analyses were done using the in-house software package Brainwave (version 0.9.152.12.26), available from http://home.kpn.nl/stam7883/brainwave.html.

The time series were digitally filtered using a discrete fast Fourier transform, to calculate the relative power for each of five canonical frequency bands: delta (0.5–4 Hz), theta (4–8 Hz), alpha (8–13 Hz), beta (13–30 Hz), and gamma (30–48 Hz) and the peak frequency (defined in the range 4–13 Hz), as a mean over 90 ROIs. PLI and AEC-c were estimated for the aforementioned five frequency bands in each cortical ROI (*n* = 78) and deep grey matter ROI (*n* = 12), and as a mean overall (*n* = 90) ROIs (referred to here as global FC). Analyses were run for each frequency band and epoch separately. The results over the 10 epochs were averaged for each subject.

#### Functional connectivity metrics

The PLI [54] is calculated from the asymmetry of the distribution of instantaneous phase differences between the time series from two brain regions, rendering it insensitive to shared signals at zero phase lag:

1 $$\mathrm{PLI}=\left|\left\langle \operatorname{sign}\left[\sin \left(\Delta \varphi \left({t}_k\right)\right)\right]\right\rangle \right|$$

where Δ*φ*(*t*_*k*_) is the phase difference at time point *t*_*k*_ between two time series, calculated for all time-points per epoch; *sign* stands for signum function; < > denotes the mean value; and || indicates the absolute value. PLI values range between 0 and 1, where 0 indicates no (non-zero-lag) coupling and 1 refers to perfect (non-zero-lag) phase locking. The phases (and amplitude envelopes) were estimated using the Hilbert transform.

The amplitude envelope correlation [55] is an amplitude-based metric which estimates the coupling between two time series by estimating the Pearson correlation between the envelopes of the amplitudes of these time series. The corrected amplitude envelope correlation (AEC-c) overcomes the effects of spatial leakage by using pair-wise orthogonalisation prior to the AEC estimation for each pair of time series (i.e. the amplitude envelopes computed using the Hilbert transform, after band-pass filtering of the signals). The correction is performed by orthonogolization separately for each pair of time/series (i.e. the power envelopes, computed using the Hilbert transform, after band-pass filtering of the signals) in two directions by means of linear regression, meaning time-series *X* is regressed out from time-series *Y* and time-series *Y* is regressed out from time-series *X*, and the AEC values (Pearson’s correlation between the orthogonalized envelopes) for both directions are averaged [38]. A value of 1 was added to all AEC-c values and then divided by two in order to obtain values in the range [0 1].

The pair-wise correlations for all combinations of ROI time series were computed for the AEC-c and PLI in each band, resulting in a symmetric 90 × 90 connectivity matrix, and subsequently averaged over rows (i.e. resulting in 1 connectivity value for each ROI, denoting the connectivity strength of that ROI with the rest of the brain).

### Statistical analyses

Normality of the variables was checked by histograms and Q-Q plots (IBM SPSS Statistics, version 26), and if violated, they were log-transformed. ANOVA on ranks [56] was performed for variables that could not be successfully log-transformed. Demographic differences and epoch quality between the AD and SCD groups, as well as the test and validation cohorts, were analysed using independent samples *t*-tests, Mann-Whitney *U* tests, or chi-square tests where appropriate. A *p* < 0.05 was considered statistically significant.

#### Sensitivity

To evaluate which FC metric and which bands were most sensitive to group differences between AD and HC subjects, we created general linear models (GLMs). GLMs were chosen as they give an estimate of the effect size (standardized beta). As mentioned in the ‘Introduction’ section, due to inconsistencies and variability in reported FC group differences between AD and controls, our main aim was to evaluate whether it was possible to obtain results that would reproduce in a second cohort (reproducibility, see the ‘Reproducibility’ section), with sufficiently large effect size (sensitivity). Additionally, this facilitated comparison with later models in which covariates were added. The FC metric (AEC-c and PLI in each band) was added as a dependent variable, and the diagnosis was entered as a predictor. In order to rule out the effect of possible neurodegeneration on our findings, the analysis was repeated excluding SCD subjects without biomarkers. To evaluate whether the observed group differences were region-specific, a Mann-Whitney *U* test was performed for the FC value in each 90 ROIs separately. False discovery rate (FDR) [57] correction was applied to *p*-values of the regional Mann-Whitney *U* group comparisons.

#### Reproducibility

To evaluate the reproducibility of the observed global differences *between* the groups using each metric, the GLMs were repeated in the validation cohort. In order to investigate whether the main results did not simply represent a ‘lucky draw’ that did not replicate to other splits of the cohort, we repeated the global analysis, splitting the sample randomly for five additional iterations, and tested whether reproducibility between the two subsets remained. FC values and effect sizes were averaged over all samples. Regionally, we repeated the Mann-Whitney *U* tests in the validation cohort to assess reproducibility. To assess the reproducibility of functional connectivity matrices *within* the patient groups (i.e. between AD subjects in the test and validation cohort), correlations were calculated using Spearman’s rho (Matlab R2012a, version 7.14.0.739), between the test and validation cohort for both FC metrics and in each band. Only the upper triangle of the symmetric FC matrices was used, and again FDR correction was applied.

#### Effects of covariates

Two GLMs were constructed to examine the effect of covariates age and the relative power of the frequency band for which the functional connectivity was estimated. Age was selected as a covariate because SCD subjects tended to be younger than AD patients. Relative power was added to evaluate the possibility that observed group effects were (partly) correlated to spectral information. Since adding covariates in a model reduces statistical power, the two cohorts were combined into one cohort to retain statistical robustness.

#### Correlation with global cognition

Finally, correlations between the global FC metrics for each band and the MMSE score were estimated using Spearman’s rho. We selected MMSE because it is a general estimate of cognitive functioning that is widely used in dementia research. The correlations were only estimated in the AD group of the test and validation cohort combined, in order to avoid inflated correlations due to the diagnosis effect.

## Results

### Cohort characteristics

The initial sample consisted of 118 subjects. Five subjects were excluded: 2 AD subjects of whom no biomarker information was available, 1 SCD subject positive for AD pathology, and 2 SCD subjects with an unreliable SCD diagnosis due to concomitant disease (epilepsy, elaborate psychiatry). The final sample consisted of 113 subjects, randomly split over two cohorts. The test cohort consisted of 29 SCD subjects and 28 AD subjects, and the validation cohort consisted of 27 SCD and 29 AD subjects. Because these cohorts were derived from clinical practice, biomarker confirmation was unavailable for 10 SCD subjects (3 in the test cohort, 7 in the validation cohort) due to failure or unwillingness to undergo lumbar punction and/or PET scan. Five of these subjects received at least one clinical follow-up, where the SCD diagnosis was upheld. Biomarker confirmation was available for all AD patients.

Within both the test and validation cohorts, the SCD groups were significantly younger than the AD groups. Additionally, the groups differed significantly (by design) on average MMSE score, amyloid positivity, CSF Aβ 1–42, CSF t-tau, and p-tau levels (see Table 1).

**Table 1:** Baseline cohort characteristics

Baseline characteristics	Test cohort (*n* = 57)		Validation cohort (*n* = 56)	
	SCD (*n* = 29)	AD (*n* = 28)	SCD (*n* = 27)	AD (*n* = 29)
Age (years)	56.7 ± 8.4	66.3 ± 7.4*	56.3 ± 10.6	64.0 ± 6.8*
Sex (female, (%))	11 (38)	16 (57)	11 (41)	16 (55)
Symptom duration (years)^a^	3.5 ± 2.6	3.3 ± 2.3	3.3 ± 2.4	2.7 ± 1.6
Education level (median, (IQR))^b^	6 (3–7)	5 (3–7)	5 (3–7)	5 (3–7)
MMSE score (median, (IQR))	28 (26–30)	20 (12–28)*^†^	27 (24–30)	22 (15–29)*†
*Cerebrospinal fluid*	***n = 19***	***n = 23***	***n = 18***	***n = 26***
Aβ 1–42 (mean ± SD)	1250 ± 159	544 ± 122*	1158 ± 224	568 ± 112*
t-tau (mean ± SD)	302.1 ± 92.0	831.4 ± 465.0*	295.6 ± 176.4	661.9 ± 337.2*
p-tau (mean ± SD)	48.9 ± 11.7	96.6 ± 37.3*	46.2 ± 12.3	81.2 ± 28.2*
*Amyloid-PET*	***n = 16***	***n = 14***	***n = 5***	***n = 7***
Positive PET (%)	0%	100%	0%	100%
Epoch quality (median, IQR)	2 (1–3)	2 (1–3)	2 (1–3)	2 (1–3)

*n.a.* not available

^a^Time between the start of symptoms and diagnosis

^b^Education level according to Verhage score (range 1–7). Depicted are the mean values ± SD or median with the interquartile range (IQR) where appropriate

^*^Significant (*p* < 0.05) group differences between AD and SCD

^†^Significant (*p* < 0.05) group differences between the test and validation cohorts

Between the two cohorts, significant differences were found between the MMSE scores; the test AD group had a lower average score than the validation AD group (median (IQR) 20 (12–28); 22 (15–29); *p* = 0.010 respectively) (see Table 1). No other differences were found between the cohorts. Importantly, the quality score of the selected epochs did not differ between both groups within the cohorts, nor between the cohorts.

### Global

#### Global sensitivity

Global FC differences between AD and SCD subjects in the test cohort can be found in Table 2. The analysis excluding the subjects without biomarkers can be found in Table S2. For the AEC-c, the AD group showed significantly higher functional connectivity than the SCD group in the delta band (mean ± SD 0.517 ± 0.013; 0.509 ± 0.007; *p* = 0.007, respectively). In contrast, compared to the SCD group, the AD group had significantly lower FC in the alpha (mean ± SD 0.521 ± 0.014; 0.530 ± 0.019; *p* = 0.038 ) and beta (mean ± SD 0.514 ± 0.006; 0.524 ± 0.017; *p* = 0.004) bands. For the PLI, significant group differences were found in the delta frequency band; however, contrary to the AEC-c, the AD group showed lower functional connectivity than the SCD group (0.111 ± 0.004; 0.113 ± 0.003; *p* = 0.038). The PLI was higher in the theta band and lower in the beta band for AD patients compared to the SCD group (0.101 ± 0.005; 0.098 ± 0.004; *p* = 0.013 and 0.052 ± 0.002; 0.053 ± 0.003; *p* = 0.026, respectively).

**Table 2:**
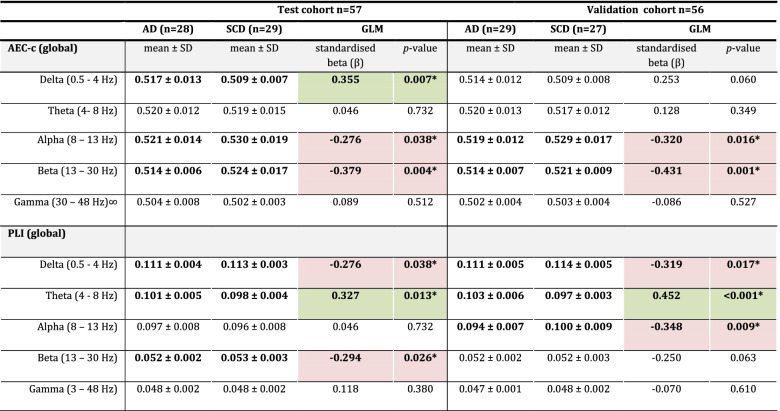
Global analysis

The difference in functional connectivity (FC) between SCD and AD subjects as estimated by GLM, for the test and validation cohorts. Shown are the mean FC values with standard deviations and the effect sizes as represented by the standardized beta. Green depicts a positive effect size, while red depicts a negative effect size

*AD* Alzheimer’s disease, *SCD* subjective cognitive decline, *GLM* general linear model, *AEC-c* corrected amplitude envelope correlation, *PLI* phase lag index, *SD* standard deviation

The AEC-c in the gamma band was analysed using ANOVA on ranks

^*^Bold print indicates a significant (*p* < 0.05) group difference

The effect sizes were small to medium for both the AEC-c and PLI, with the largest effect sizes in the beta band for the AEC-c test and validation cohort (*β* = − 0.379; *β* = − 0.431, respectively) and in the theta band for the PLI test and validation cohort (*β* = 0.327; *β* = 0.452, respectively).

#### Global reproducibility

When repeating the analyses in the validation cohort, the group differences remained significant for the AEC-c alpha and beta band (Table 2). Similarly, the PLI again showed significant group differences for the delta and theta bands. While no significant group difference was found for the beta band PLI for the validation cohort, a statistically significant difference was observed in the alpha band with lower PLI for the AD group (0.094 ± 0.007; 0.100 ± 0.009; *p* = 0.009) compared to the SCD group. The results for the additional splits can be found in the Supplementary Information Table S3. The AEC-c beta band was consistently reproducible across all six splits. Four out of six splits gave reproducible differences for the PLI theta band; the AEC-c alpha band reproduced in three out of six splits. Finally, both the AEC-c and PLI delta band were reproducible across two splits. The other FC measures consistently did not reproduce.

### Regional

#### Regional sensitivity and reproducibility

**Fig. 1:**
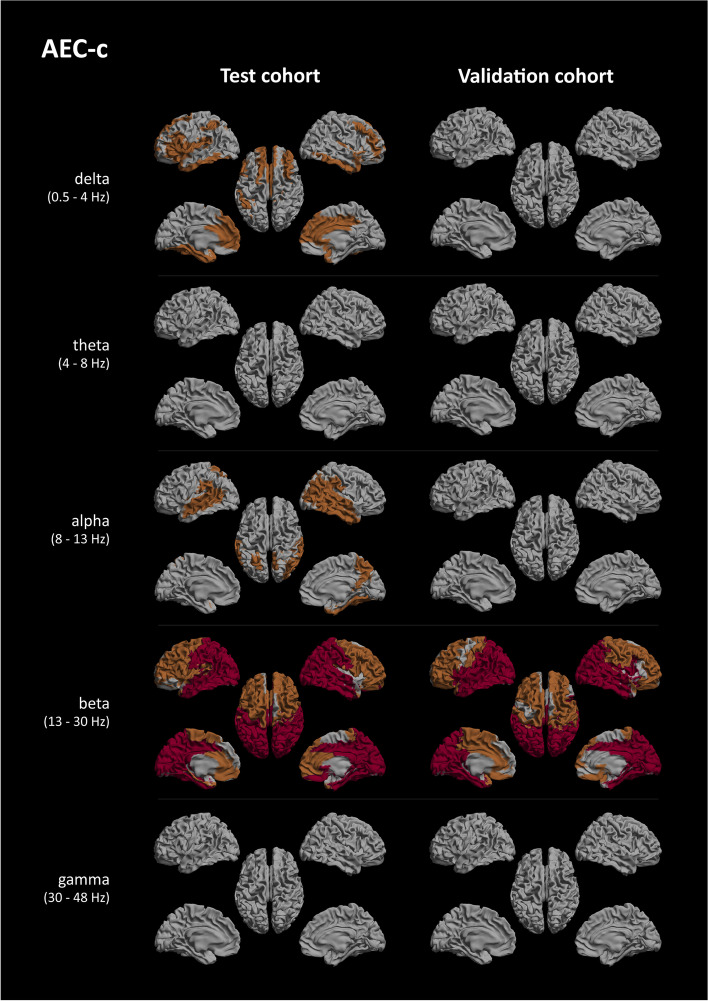
AEC-c significant regional group differences. Regions of interest where significant group differences, as determined using Mann-Whitney *U* testing and FDR-corrected for multiple comparisons, between the AD and SCD groups were found, shown as a colour-coded map on a template mesh. Each row represents a different frequency band (delta, theta, alpha, beta, and gamma), and the columns show the results for the test cohort (left) and validation cohort (right). Orange indicates *p* < 0.05, and red indicates *p* < 0.01

The regions with significant FDR-corrected group differences for both FC measures can be seen in Fig. 1 for AEC-c and 3 for PLI. The results uncorrected for multiple comparisons can be found in Supplementary Figs. S1, S2, and S3 and are further discussed in the Supplementary Information.

**Fig. 2:**
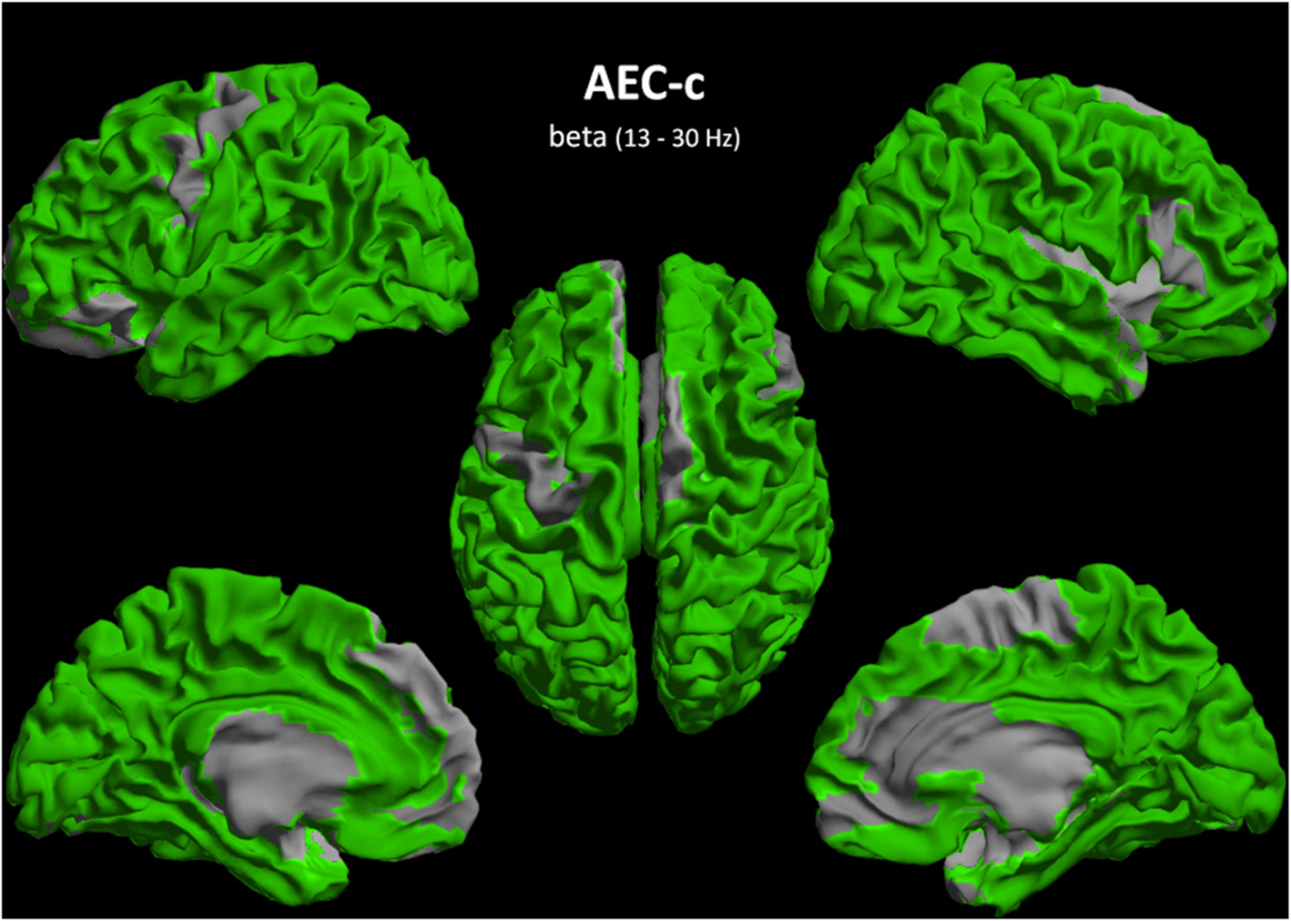
Reproducibility of regional group differences AEC-c beta band. A colour-coded map on a template mesh showing the reproducibility of significant group differences as overlapping regions of interest between the test and validation cohorts, as determined using the Mann-Whitney *U* tests and FDR-corrected for multiple comparisons, shown in green colour. See also Fig. 1

For the AEC-c beta band, FDR-corrected group differences were found in almost every ROI for the test and validation cohort, except for 13 ROIs in the frontal and temporal lobes (for specific details see Table S4). The most reproducible region was the occipital lobe, more specifically all occipital ROIs revealed highly (*p* < 0.01) significant group differences in both cohorts, with the parietal lobe as the second most reproducible. AD-related regions, such as the left and right precuneus, left and right hippocampi, and the cingulate gyri, were equally highly reproducible, with the exception of the left and right anterior cingulate (Figs. 1 and 2, Table S4). Of the subcortical regions, the hippocampi and right thalamus were reproducibly highly significant, although all subcortical regions showed reproducible group differences.

For the AEC-c alpha band, after FDR correction, the significant group differences in the validation cohort disappeared, such that the significant alpha band results in the test cohort did not reproduce. A similar effect was seen for the delta band, where all regions in the validation cohort lost significance after FDR correction, and therefore, the results from the test cohort did not reproduce.

Both the AEC-c theta and gamma bands displayed no overlap between the cohorts.

**Fig. 3:**
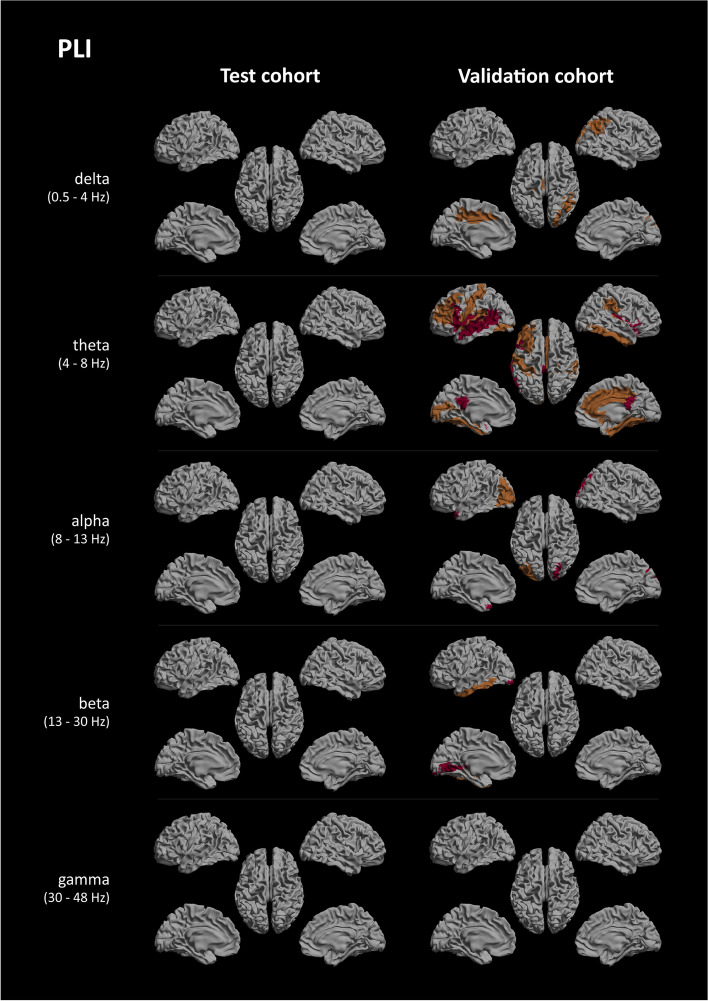
PLI significant regional group differences. Regions of interest where significant group differences, as determined using Mann-Whitney *U* testing and FDR-corrected for multiple comparisons, between the AD and SCD groups were found, shown as a colour-coded map on a template mesh. Each row represents a different frequency band (delta, theta, alpha, beta, and gamma), and the columns show the results for the test cohort (left) and validation cohort (right). Orange indicates *p* < 0.05, and red indicates *p* < 0.01

After FDR correction, the PLI showed no reproducible regional group differences for any bands (see Fig. 3). For the uncorrected results, see again the Supplementary Information.

#### Connectivity matrices

**Fig. 4:**
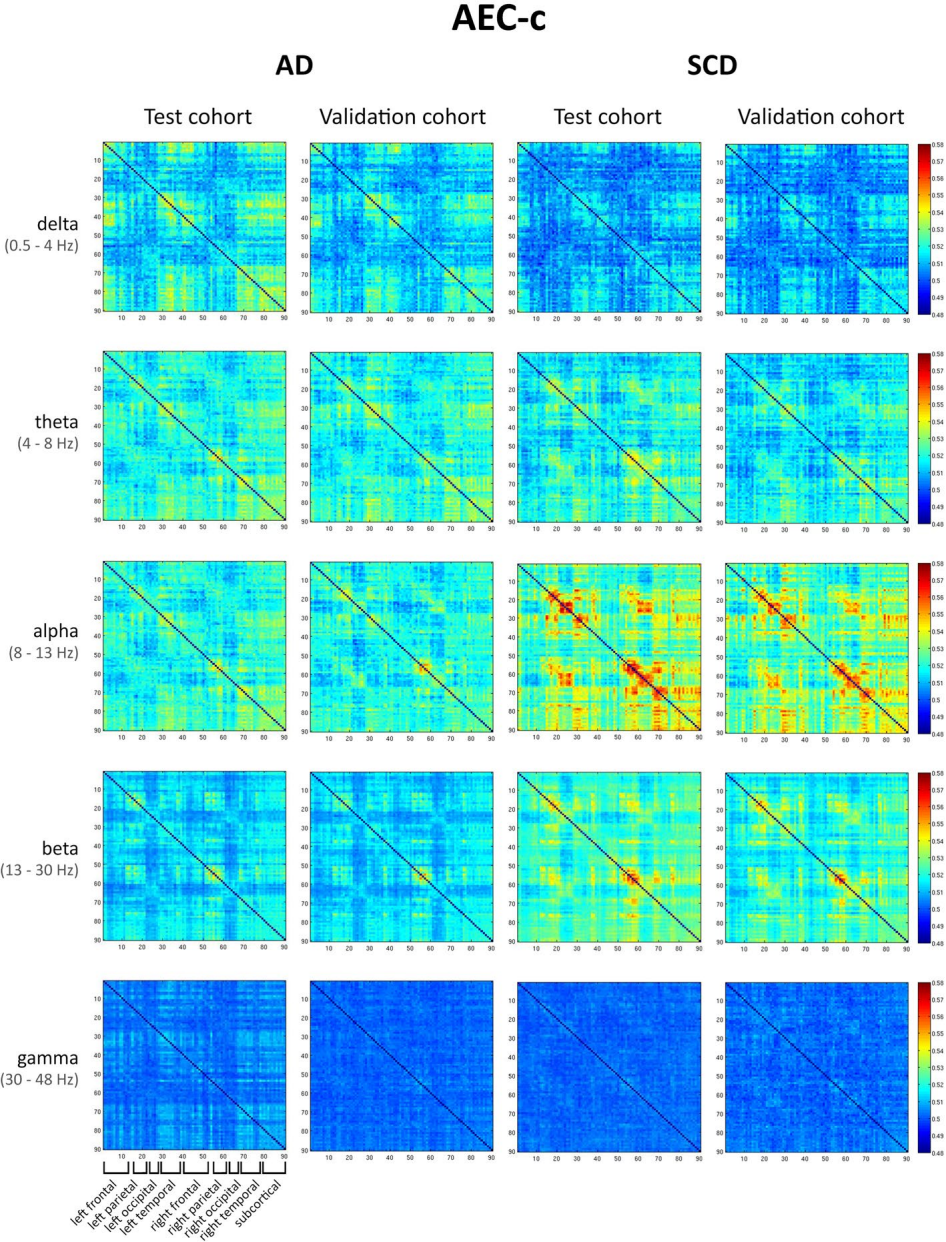
Connectivity matrices of AEC-c. Connectivity matrices averaged across all epochs and subjects. Each row represents a different frequency band (delta, theta, alpha, beta, and gamma), and each column shows the results for the AEC-c, for the test and validation cohorts in the AD and SCD groups. All bands and groups show the matrices with the same scale. The ROIs are obtained from the AAL atlas. The matrices are ordered from the left to the right hemisphere in the following way: rows/columns 1–15 represent left frontal regions, 16–21 left parietal regions, 22–27 left occipital regions, 28–39 left temporal regions, 40–54 right frontal regions, 55–60 right parietal regions, 61–66 right occipital regions, 67–78 right temporal regions, and 79–90 subcortical regions (see also Table S1)

**Fig. 5:**
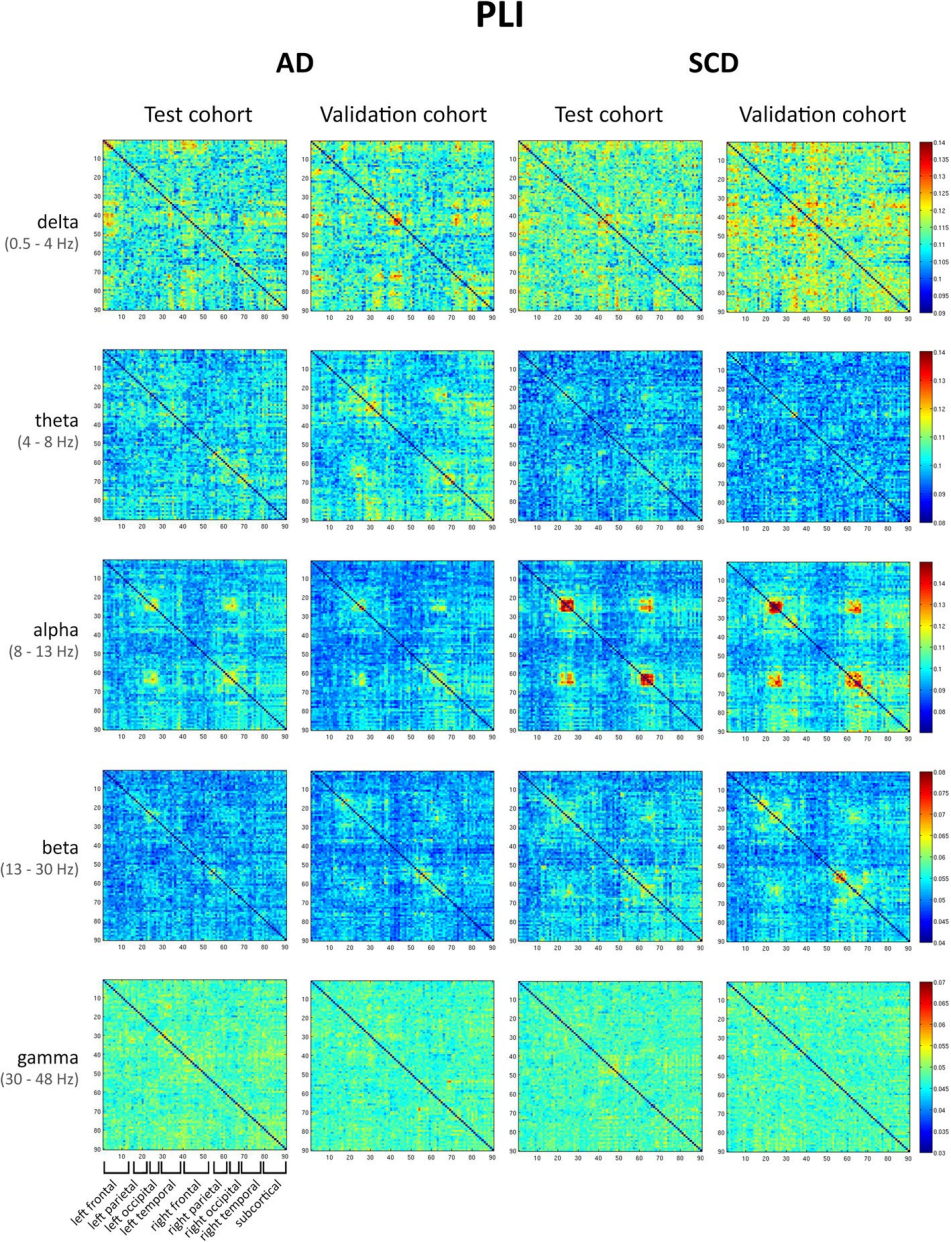
Connectivity matrices PLI. Connectivity matrices averaged across all epochs and subjects. Each row represents a different frequency band (delta, theta, alpha, beta, and gamma), and each column shows the results for the PLI, for the test and validation cohorts in the AD and SCD groups. Note that in this figure, the scale is different for each band but the same across the groups. The ROIs are obtained from the AAL atlas, ordered from the left to the right hemisphere, see Fig. 4 and Table S1 for a more detailed description. See also Fig. S4 for the same results displayed using the same colour scale

Figure 4 shows the average FC matrices for every frequency band for the AEC-c and for the AD and SCD groups in the test and validation cohorts. Figure 5 shows the resultant matrices for the PLI with a different colour scale for each band, because visual inspection of the matrices was hampered when using a similar colour scale across bands. The matrices with the same colour scale across bands can be found in Fig. S4.

On visual inspection, the matrices obtained with the AEC-c showed high reproducibility of the FC pattern for both the AD and SCD groups, for all bands except gamma. The AEC-c SCD groups showed a strongly reproducible FC pattern in the alpha band (and to some extent in the beta band), which was absent in the AD test and validation cohorts. Instead, the AD groups showed a more pronounced connectivity pattern in the delta band. In the SCD alpha band, especially the parietal regions showed higher values for off-diagonal entries corresponding to FC between homologous ROIs (left parietal connects with right parietal). Quantifying FC reproducibility on the matrix level, the correlation between matrices from the test and validation cohort was highest in the beta band for the AD group (*r*_*s*_ (88) = 0.87; *p* < 0.001, see also Table 3) and alpha band for the SCD group, although the beta band also performed well (*r*_*s*_ (88) = 0.87; *p* < 0.001 and *r*_*s*_ = 0.82 (88); *p* < 0.001 respectively). For both groups, the gamma band showed the lowest correlations.

**Table 3:** Correlations between connectivity matrices

AEC-c				PLI			
AD		SCD		AD		SCD	
*Delta (0.5–4 Hz)*	***r***_***s***_ **= 0.74** ***p*** < 0.001*	*Delta (0.5–4 Hz)*	***r***_***s***_ **= 0.60** ***p*** < 0.001*	*Delta (0.5–4 Hz)*	***r***_***s***_ **= 0.13** ***p*** < 0.001*	*Delta (0.5–4 Hz)*	***r***_***s***_ **= 0.11** ***p*** < 0.001*
*Theta (4–8 Hz)*	***r***_***s***_ **= 0.70** ***p*** < 0.001*	*Theta (4–8 Hz)*	***r***_***s***_ **= 0.71** ***p*** < 0.001*	*Theta (4–8 Hz)*	***r***_***s***_ **= 0.23** ***p*** < 0.001*	*Theta (4–8 Hz)*	***r***_***s***_ **= 0.09** ***p*** < 0.001*
*Alpha (8–13 Hz)*	***r***_***s***_ **= 0.62** ***p*** < 0.001*	*Alpha (8–13 Hz)*	***r***_***s***_ **= 0.87** ***p*** < 0.001*	*Alpha (8–13 Hz)*	***r***_***s***_ **= 0.41** ***p*** < 0.001*	*Alpha (8–13 Hz)*	***r***_***s***_ **= 0.49** ***p*** < 0.001*
*Beta (13–30 Hz)*	***r***_***s***_ **= 0.87** ***p*** < 0.001*	*Beta (13–30 Hz)*	***r***_***s***_ **= 0.82** ***p*** < 0.001*	*Beta (13–30 Hz)*	***r***_***s***_ **= 0.21** ***p*** < 0.001*	*Beta (13–30 Hz)*	***r***_***s***_ **= 0.37** ***p*** < 0.001*
*Gamma (30–48 Hz)*	***r***_***s***_ **= 0.42** ***p*** < 0.001*	*Gamma (30–48 Hz)*	***r***_***s***_ **= 0.25** ***p*** < 0.001*	*Gamma (30–48 Hz)*	***r***_***s***_ **= 0.06** ***p*** < 0.001*	*Gamma (30–48 Hz)*	*r*_*s*_ = 0.03 *p* = 0.0910

Spearman’s rho coefficients (d.f. 88) correlating the connectivity matrices for the test and validation cohorts, for the AD and SCD groups. On the left side, the results for the AEC-c are displayed, and on the right, the results for the PLI

^*^Bold print depicts a significant (*p* < 0.05) correlation

For matrices computed using the PLI, a reproducible within-group pattern was found for the alpha and beta band, for both patient groups. The delta, theta, and gamma bands showed low reproducibility between the test and validation cohorts. This is reflected by the correlations between matrices from the two cohorts, where for both AD and SCD groups, the alpha band had the highest correlation coefficients (*r*_*s*_ (88) = 0.41; *p* < 0.001 and *r*_*s*_ (88) = 0.49; *p* < 0.001 respectively, see Table 3), although the correlation strength was lower than for the AEC-c. The delta and theta bands showed a higher correlation for AD than for SCD, although correlations were weak. The gamma band again performed worst for both groups.

### Influence of covariates (age/relative power)

We repeated the GLM without covariates on the combined test and validation cohort, creating a pool of 57 AD patients and 56 SCD subjects. In the combined model without covariates, we confirmed significant group differences for the AEC-c in the delta, alpha, and beta bands (see also Table 4). For the PLI, we found significant differences for the delta, theta, and beta bands.

**Table 4:**
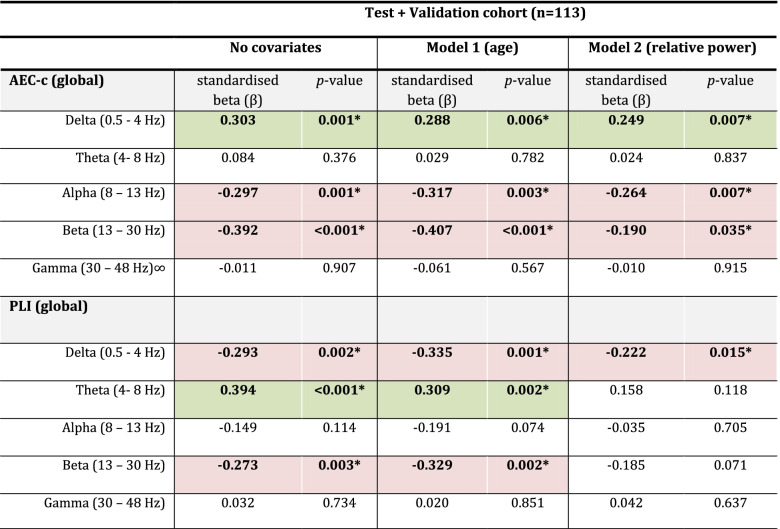
Covariates

The difference in functional connectivity (FC) between SCD and AD subjects after correcting for age (model 1) and relative power (model 2). The AEC-c in the gamma band was analysed using ANOVA on ranks

Green depicts a positive effect size, while red depicts a negative effect size

^*^Bold print represents a significant group difference

To investigate whether the observed group differences were influenced by the age differences between the AD and SCD groups, a second GLM with age as a covariate was created (see again Table 4). Adding age did not significantly influence the results.

A third model with relative power in the corresponding frequency band as a covariate was created. For the AEC-c, all results remained significant. For the PLI, the delta band result remained significant (*β* = − 0.222, *p* = 0.015), but the theta and beta bands lost significance (*β* = 0.158, *p* = 0.118; *β* = − 0.185, *p* = 0.71, respectively).

### Correlation with cognitive performance

Spearman’s rho values were calculated between the AEC-c/PLI and the MMSE score for the combined AD group (*n* = 55, as there were two missing values for MMSE scores). Only the AEC-c in the alpha band showed a significant positive correlation with cognitive functioning (*r*_*s*_ (53) = 0.309, *p* = 0.022), see also Table 5.

**Table 5:** Correlation with cognition (MMSE)

Versus MMSE	AD (*n* = 55)	
	***r***_***s***_ (53)	***p***-value
AEC-c		
Delta (0.5–4 Hz)	− 0.104	0.451
Theta (4–8 Hz)	0.175	0.201
Alpha (8–13 Hz)	**0.309**	0.022*
Beta (13–30 Hz)	0.262i	0.053
Gamma (30–48 Hz)	− 0.012	0.932
PLI		
Delta (0.5–4 Hz)	0.178	0.193
Theta (4–8 Hz)	− 0.010	0.945
Alpha (8–13 Hz)	0.250	0.066
Beta (13–30 Hz)	− 0.010	0.943
Gamma (30–48 Hz)	0.043	0.753

Spearman’s rho coefficients (d.f. 53) correlating functional connectivity to cognition as represented by MMSE (*n* = 55)

^*^Bold print depicts a significant (*p* < 0.05) correlation

## Discussion

The aim of this study was to identify which functional connectivity metrics could reproduce a given pattern of FC differences, in two clinically derived MEG-cohorts of patients with AD dementia and cognitively healthy control subjects. We specifically focused on two measures that are insensitive to the effects of volume conduction/field spread: the AEC-c and the PLI.

Our most important finding is that reproducible differences were found for the global AEC-c in the alpha and beta bands and for the global PLI in the delta and theta bands. The AEC-c effects were independent of relative power and age, and the alpha band AEC-c correlated with MMSE scores. For the PLI, the theta band was independent to the effect of age, but this was not the case for relative band power. The high spatial resolution of MEG compared to EEG, allowed for the exploration of group differences on a regional level, which showed reproducible effects in almost all ROIs for the AEC-c in the beta band, and also in AD-specific regions such as the precunei, the hippocampi, and the cingulate gyri. Regionally, the PLI showed no reproducible group differences after FDR correction. Finally, connectivity matrices were highly reproducible within patient groups, with especially high correlations (*r*_*s*_ (88) 0.82–0.87) for the AEC-c beta band.

### Global FC

The results of the global analysis show the AEC-c to be a consistent metric when it comes to group-level reproducibility. Even across additional samples, only the AEC-c beta band was consistently reproducible across all six splits, while the AEC-c alpha band reproduced three out of six splits. This is in line with previous studies that identified the AEC-c as having high test-retest reliability [27, 28], specifically in the alpha and beta bands [38, 58]. Our study extends upon this previous work by evaluating the reproducibility of AEC-c differences between AD dementia and SCD in two independent cohorts, rather than a test-retest design of one patient group. We observed consistent decreases in AEC-c for the alpha and beta bands in AD dementia compared to SCD; these findings are again supported by previous MEG [11] and EEG [24, 30, 59] studies. Another previous study compared subjects with mild cognitive impairment (MCI) to elderly controls, using uncorrected MEG-derived envelope amplitude correlations as FC metric and structural connectivity based on diffusion imaging, and found that both functional connectivity in the alpha band and structural connectivity were decreased for the MCI compared to the control group [60]. Our current results are therefore in line with previous findings that AD is associated with a decrease in amplitude-based functional coupling of alpha and beta rhythms. Importantly, we have demonstrated previously in an EEG study, which evaluated the reproducibility of several connectivity measures in a cohort of SCD versus AD subjects, that these findings are specific for Alzheimer’s disease using a population subset specifically selected on the presence of Alzheimer’s pathology [24]. The effect sizes in our study were also comparable to this study, which found an effect size of − 0.5 for the AEC-c alpha and − 0.4 for the AEC-c beta band (compared to our ~ − 0.3 and − 0.4, respectively).

On a global level, higher PLI was observed in the theta band for the AD group, which was reproduced in the independent validation cohort. When evaluating whether our results would replicate to other splits of the cohort, four out of six splits gave reproducible differences for the PLI theta band. A previous high-density EEG study has shown good reliability of the global theta band PLI (with an intraclass correlation (ICC) of 0.72), although it was outperformed by the alpha 1 and 2 bands [61]. Importantly, this study examined test-retest reliability and did not focus on group differences. In our study, the PLI alpha band did not reproduce across splits. In another source-space test-retest MEG study, the PLI yielded low ICC over frequency bands in healthy subjects, including the theta band [58]. In previous AD literature, PLI-derived functional connectivity in AD patients has been characterized by an increase in the theta band, with a widespread decrease in alpha and beta band connectivity, especially in later stages [9, 32, 33]. Another study compared MCI subjects to healthy controls using another phase-based measure, the phase-locking value (PLV), and detected a parieto-occipital increase in connectivity in the theta band for the MCI group compared to the control group [62]. Although we did find subtly lower mean beta band FC for the AD group of the test cohort, this did not reproduce in the validation cohort. Vice versa, we found a subtly lower alpha band FC in the validation cohort which was not present in the test cohort. Importantly, Briels et al. also identified the theta band as most robust for the PLI (in sensor-level EEG), although, similar to our study, the PLI was not independent of changes in relative power. The effect size was also comparable to our findings; 0.6 for the PLI theta band, compared to 0.4 in our study.

An interesting observation is that the AEC-c and PLI found opposite group effects in the delta band; while the global AEC-c increased for the AD group compared to controls, the global PLI decreased. This phenomenon could also be observed visually for many of the individual connections, i.e. in the connectivity matrices. A possible explanation is the sensitivity to different characteristics of the oscillatory signal (amplitude-based versus phase-based), with phase coupling playing a role in neural communication on faster temporal scales than envelope correlations, which may be more involved in preparing neural populations for input [5]. Both measures likely play an important role in establishing functional networks, capturing different aspects of functional connectivity and possibly cognition [5]. The phase of the oscillations is thought to represent the degree of excitability of neuronal populations, while the amplitude represents the intensity of coherent neuronal activity [58]. Additionally, previous studies suggest that power envelope correlations are more linked to the underlying structural connectome [5]. Therefore, the PLI decrease and AEC-c increase for the delta band could represent different aspects of network pathology in AD. Whether amplitude- and phase-based connectivity are two interacting modalities or are sensitive to different disease effects is as of yet uncertain, although recent work suggests that they may reflect at least partially distinct neuronal mechanisms [63]. Possibly, these two measures might be sensitive to disease effects at different stages of the trajectory, with phase synchrony changes in earlier stages compared to amplitude correlation changes [36]. While the current study is unable to answer this question, this is an interesting consideration for future work***.***

After removing SCD subjects without biomarkers from the analysis, most results were very similar between the two analyses (Table S2); however, the AEC-c alpha band group difference lost significance in both cohorts. Interestingly, the exclusion of the subjects without biomarkers resulted in significant (and therefore now reproducible) group differences in the PLI beta band. A possible explanation might be that some of the SCD subjects without biomarker information do contain some neurodegeneration; if such subjects were present, they may have already shown some phase disruption, which may have originally impacted our ability to detect group differences in phase-based connectivity in this band. However, the AEC-c delta band also became reproducible. Importantly, excluding the SCD subjects without biomarkers constitutes an almost 10% loss of power, allowing for limited conclusive statements.

### Regional FC

In addition to the global analysis, the AEC-c beta band showed high regional consistency. Group differences were found for almost the entire cortex and subcortical areas, with some exceptions in the frontal and temporal regions. The most highly significant reproducible group differences (Fig. 1) were found in the occipital and parietal regions. A previous MEG study that used a whole-brain voxel-based analysis found significantly decreased beta band AEC-c in AD compared to elderly controls, which was mostly localized to bilateral inferior parietal and superior temporal areas [11]. Importantly, our study has shown for the first time that the AEC-c beta band can be used to measure FC differences on both a cortical and subcortical level. Notably, reproducibility was excellent in AD-related areas such as the precunei, hippocampi, and the cingulate gyri. For the AEC-c alpha band, while regional group differences remained significant after FDR correction in the test cohort, this was not the case for the validation cohort, resulting in poor reproducibility.

Looking at the extent of reproducible regional group differences between test and validation cohort, we expected the PLI theta band results to remain significant after FDR correction. However, this was not the case. Previous work in healthy controls showed a strong dominance in the parieto-occipital regions for the PLI, in accordance with the topology of structural and functional connectomes derived from MRI studies [61].

An important fact to note is that because of our focus on identifying reproducible group differences, we only looked at true positives; true negatives were thus left out of consideration. The results in for instance both the AEC-c and PLI gamma bands, which consistently showed no (reproducible) group differences, must therefore not necessarily be interpreted as poor performance.

Analysis of the connectivity matrices yields three important conclusions: firstly, the patterns of strong AEC-c connections showed more spatial structure than the PLI, which have a noisier appearance. In the alpha band for both the AEC-c and PLI SCD group, the ‘four-block’ structure, which corresponds to higher connectivity between parietal and occipital ROIs and between homologous parietal and occipital ROIs, can be seen, although it is more pronounced for the AEC-c. Possibly, as the PLI might be sensitive to neuronal communication on a faster temporal scale, this leads to more variability within an epoch and therefore lower PLI values. It might therefore be preferable to look at dynamic phase-based FC [30, 64]. Secondly, matrices were highly reproducible within groups, across two separate cohorts. Thirdly, both the AEC-c and PLI show a widespread loss of functional connectivity in the alpha and beta band for AD. Comparing AD with SCD, the loss of FC in the alpha band and increase in FC in the lower frequency bands can be observed for both measures, although for the AEC-c, it seems more pronounced in the delta band, and for the PLI in the theta band. Alzheimer’s disease is often considered to be a ‘disconnection syndrome’, characterized by a loss of network integrity and altered synchronizability in the higher frequency bands [10, 23, 65]. Our results provide important evidence for widespread loss of connectivity in AD.

### Influence of relative power

The effects observed in the AEC-c bands remained stable after correcting for relative power, while the theta band findings for the PLI disappeared; these findings are comparable to a previous EEG study [59]. The fact that the AEC-c has robustly shown itself to be independent of the effects of relative power provides evidence that the AEC-c can reliably be used as an independent measure of connectivity in AD and SCD, whether jointly or separate from relative power. This does not, however, automatically disqualify the PLI. Importantly, previous work by Tewarie and colleagues suggests that functional connectivity and oscillatory activity might not be completely independent and that local modulations in neural oscillatory amplitude reflect modulations in connectivity between that region and the rest of the brain in resting-state MEG, sensorimotor task MEG, and in simulated data based on a neuronal model [64]. Statistical correction for the effect of relative theta power on metrics such as the PLI should therefore be applied with caution.

### Correlation with cognitive performance

We found that the MMSE score correlated with the alpha band AEC-c in the AD group, congruent with a previous finding by Briels and colleagues. These results were uncorrected for multiple comparisons due to their exploratory nature and should therefore be interpreted with some caution. We used MMSE as a measure of global cognition; future studies could evaluate whether these measures of FC correlate more strongly with specific cognitive domains.

### Strengths and limitations

The fact that we assessed FC sensitivity, and most importantly, reproducibility in clinical cohorts is an important strength of the current study. Most previous studies have performed a test-retest design, while the current study analyses differences between AD dementia patients and SCD in two independent cohorts. As mentioned before, the lack of research into this topic has hampered consensus on the value of FC metrics in the research setting and in clinical practice. Another important strength is the fact that biomarker proof was available for the majority of our subjects. Additionally, the high spatial resolution of MEG compared to EEG allowed us to investigate reproducibility on a regional cortical and subcortical level and zoom in on AD-related areas.

There are some potential limitations that need to be considered. Firstly, because our cohort was derived from clinical practice, biomarker confirmation was unavailable for several SCD subjects. In order to uphold statistical power, we decided not to exclude these subjects. It is therefore not impossible to rule out the presence of AD positive biomarkers in these subjects. However, we argue that this may not be a limitation as the focus of this project was to evaluate the FC metrics in a clinically representative cohort. Notably, we repeated the results by Briels and colleagues, who already showed the value of these metrics when strictly applying the A/T/N research criteria [66].

Secondly, the frontal regions in MEG are more susceptible to the presence of artefacts (e.g. eye movements), even after source reconstruction. Although we carefully selected the epochs used in the analysis, the delta band results found for frontal regions for the AEC-c test cohort should therefore be interpreted with some caution. The finding that these results could not be replicated in the validation cohort is already suggestive. Future studies should further evaluate these findings, especially since a recent MEG study provided putative evidence that the functional connectivity (as measured by imaginary coherence, an estimate of neural synchrony) in the delta band in frontal regions may indeed have clinical value in AD [67].

Thirdly, the AEC-c and PLI are insensitive to zero phase-lagged interaction (by construction) in order to reduce their sensitivity to the effects of volume conduction and field spread. As a consequence, physiological connectivity with zero phase-lag remains undetected, and the AEC-c and/or PLI may therefore have underestimated true connections.

Fourthly, another limitation is the use of template MRI’s for the co-registration. Given the variability of individual brain atrophy levels that can be observed with age, coregistration with individual MRIs would improve source reconstruction accuracy. The subcortical results may therefore need to be interpreted with some caution. However, it has been shown by our group that the use of MRI templates, instead of native MRI scans, produces reliable source-space time series without (systematic) bias with regard to MEG spectral and functional connectivity measures [68].

Finally, in the uncorrected results, a large difference was found between the number of regions that were found to reveal significant group differences in the PLI alpha band for the test cohort, compared to the validation cohort. The cohorts had similar demographic distribution, clinical progression, and epoch quality; a possible explanation might be the slight difference in MMSE score between the two AD groups, since the two cohorts were randomly allocated at the start of the study. Future studies could attempt to elucidate these results. If anything, they highlight the importance of reproducibility in science.

## Conclusion

We conclude that in two separate clinical MEG cohorts, the AEC-c is a sensitive, reproducible measure in the evaluation of FC differences between AD dementia and SCD on a global and regional level, with the beta band providing the most robust estimates of FC. The PLI was sensitive and reproducible on a global level in the theta band, but not on a regional level. Our results provide important evidence regarding the sensitivity and reproducibility of functional connectivity changes in AD.

## Supplementary Information

**Additional file 1: Fig. S1.** AEC-c significant regional group differences. Regions of interest where significant group differences, as determined using Mann-Whitney U testing (*p*<0.05, uncorrected), between the AD and SCD groups were found, shown as a color-coded map on a template mesh. Results are uncorrected for multiple comparisons. Each row represents a different frequency band (delta, theta, alpha, beta and gamma), and the columns show results for the test cohort (left) and validation cohort (right). Orange indicates *p*<0.05 and red indicates *p*<0.01 (uncorrected).

**Additional file 2: Fig. S2.** PLI significant regional group differences. Regions of interest where significant group differences, as determined using Mann-Whitney U testing (*p*<0.05, uncorrected), between the AD and SCD groups were found, shown as a color-coded map on a template mesh. Results are uncorrected for multiple comparisons. Each row represents a different frequency band (delta, theta, alpha, beta and gamma), and the columns show results for the test cohort (left) and validation cohort (right). Orange indicates *p*<0.05 and red indicates *p*<0.01 (uncorrected).

**Additional file 3: Fig. S3.** Reproducibility of regional group differences. A color-coded map on a template mesh showing reproducibility of significant group differences as overlapping regions of interest between the test and validation cohort, as determined using Mann-Whitney U tests (*p*<0.05, uncorrected), shown in green colour. See also Figures S1 and S2. Each row represents a different frequency band (delta, theta, alpha, beta and gamma), and the columns show results for the AEC-c (left) and PLI (right).

**Additional file 4: Fig. S4.** Connectivity matrices PLI with same colour scale. Connectivity matrices averaged across all epochs and subjects. Each row represents a different frequency band (delta, theta, alpha, beta and gamma), and each column shows results for the PLI, comparing the test and validation cohort in the AD and SCD groups. All bands show the matrices with the same colour scale. The ROIs are obtained from the AAL atlas, ordered from left to right hemisphere, see Figure 4 and Table S1 for more detailed description.

**Additional file 5: Table S1.** AAL atlas regions [44]. **Table S2.** Global analysis excluding control subjects without biomarkers. Difference in functional connectivity (FC) between SC and AD subjects as estimated by GLM, for the test and validation cohort, excluding subjects with missing biomarkers. Shown are the mean FC values with standard deviations, and the effect sizes as represented by the standardized beta. The AEC-c in the gamma band was analysed using ANOVA on ranks. *bold print indicates a significant (p <0.05) group difference. Print in italics and underscored represents a deviation from the results that were obtained for the analysis that included all subjects. AD: Alzheimer’s disease; SCD: Subjective cognitive decline; GLM: general linear model; AEC-c: corrected amplitude envelope correlation; PLI: phase lag index; SD: standard deviation. **Table S3.** Global analysis over 5 split-sample iterations + original sample. Difference in functional connectivity (FC) between SCD and AD subjects as estimated by GLM, for the test and validation cohort**,** for all iterations (five additional samples + original sample). Shown are the mean FC values with standard deviations, and the effect sizes as represented by the standardized beta. The AEC-c in the gamma band was analysed using ANOVA on ranks. *bold print indicates a significant (p <0.05) group difference. AD: Alzheimer’s disease; SCD: Subjective cognitive decline; GLM: general linear model; AEC-c: corrected amplitude envelope correlation; PLI: phase lag index; SD: standard deviation. ICC: Intra-class correlation. **Table S4.** FDR-corrected Mann-Whitney U outcomes for AEC-c beta band. Depicted are the regional Mann-Whitney U significance outcomes for the AEC-c in the beta band for both the test and validation cohort. The left column depicts the ROI number, while the subsequent columns depict the test outcomes. *signifies *p*<0.05, corrected.

## Data Availability

Due to the clinical nature of the data, the data that support the findings of this study are not freely available but can be made available by the corresponding author, upon reasonable request. A formal data sharing agreement is needed before any data can be shared.
