# Supplementary figures and images for: Sensitive and reproducible MEG resting-state metrics of functional connectivity in Alzheimer’s disease
Source: Alzheimers Res Ther. 2022 Feb 26;14:38. doi: 10.1186/s13195-022-00970-4 (PMC8881826; doi:10.1186/s13195-022-00970-4)

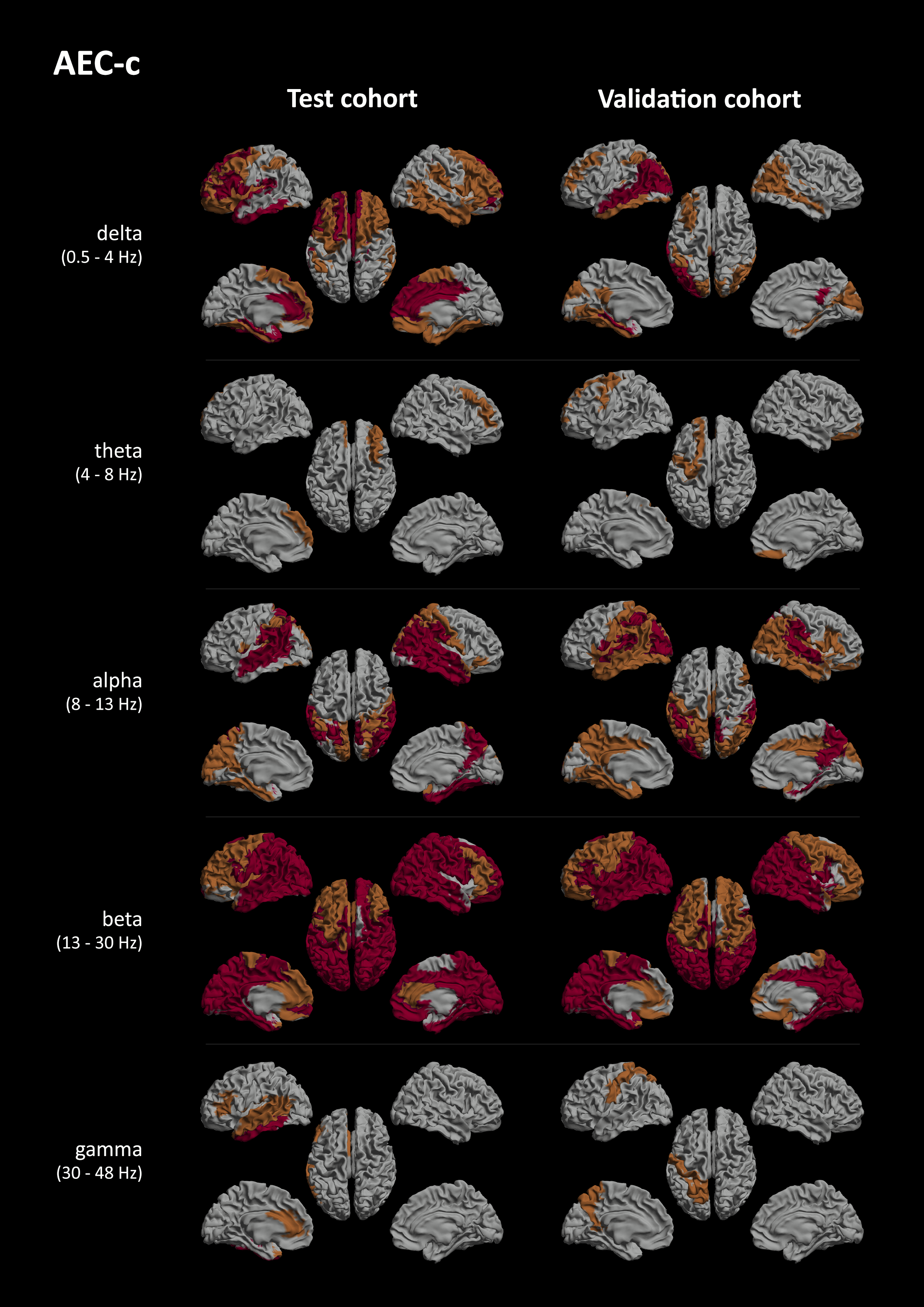

Supplement: Supplementary file 1 — Additional file 1: Fig. S1. AEC-c significant regional group differences. Regions of interest where significant group differences, as determined using Mann-Whitney U testing (p<0.05, uncorrected), between the AD and SCD groups were found, shown as a color-coded map on a template mesh. Results are uncorrected for multiple comparisons. Each row represents a different frequency band (delta, theta, alpha, beta and gamma), and the columns show results for the test cohort (left) and validation cohort (right). Orange indicates p<0.05 and red indicates p<0.01 (uncorrected). [file 13195_2022_970_MOESM1_ESM.tif]

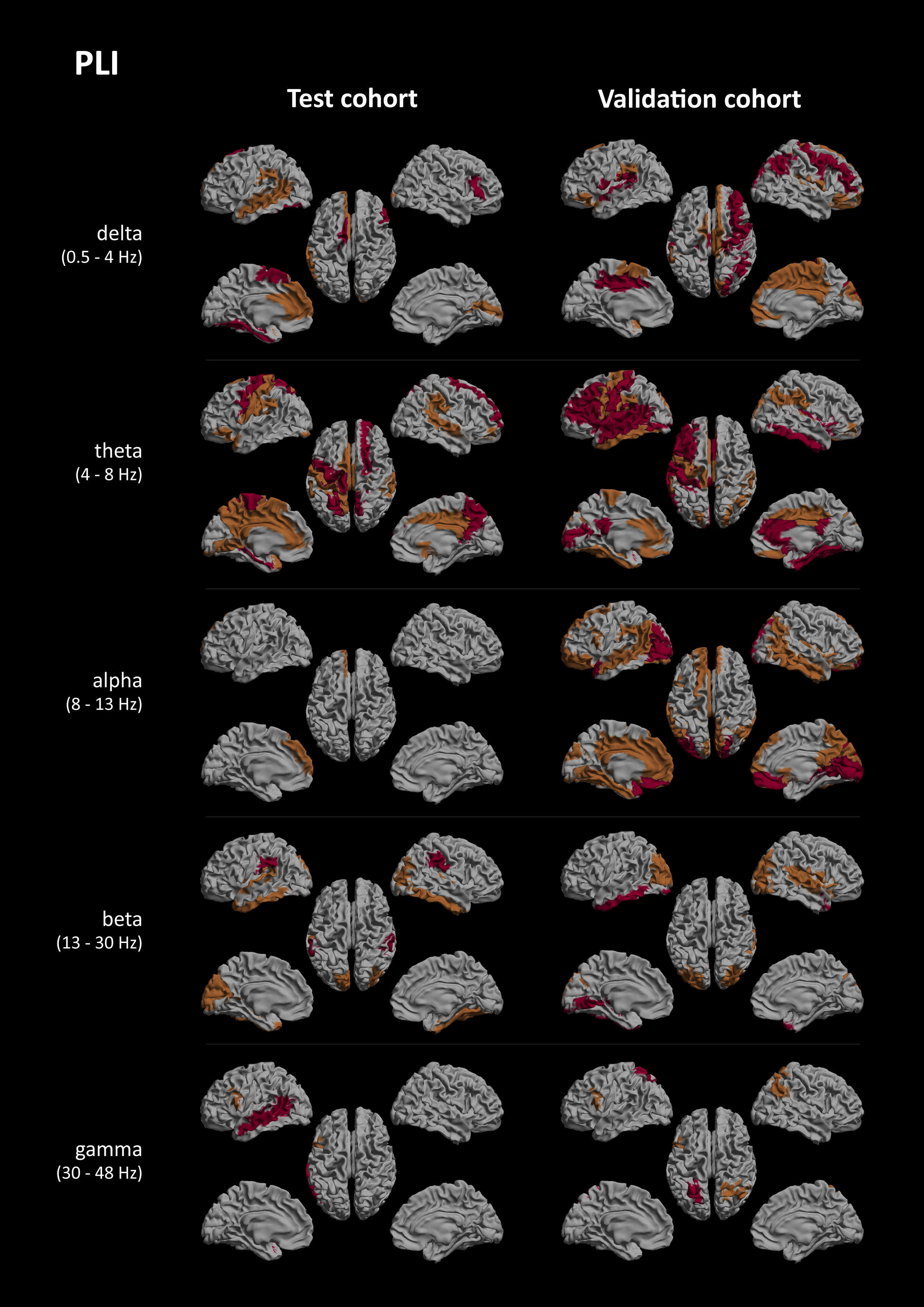

Supplement: Supplementary file 2 — Additional file 2: Fig. S2. PLI significant regional group differences. Regions of interest where significant group differences, as determined using Mann-Whitney U testing (p<0.05, uncorrected), between the AD and SCD groups were found, shown as a color-coded map on a template mesh. Results are uncorrected for multiple comparisons. Each row represents a different frequency band (delta, theta, alpha, beta and gamma), and the columns show results for the test cohort (left) and validation cohort (right). Orange indicates p<0.05 and red indicates p<0.01 (uncorrected). [file 13195_2022_970_MOESM2_ESM.tif]

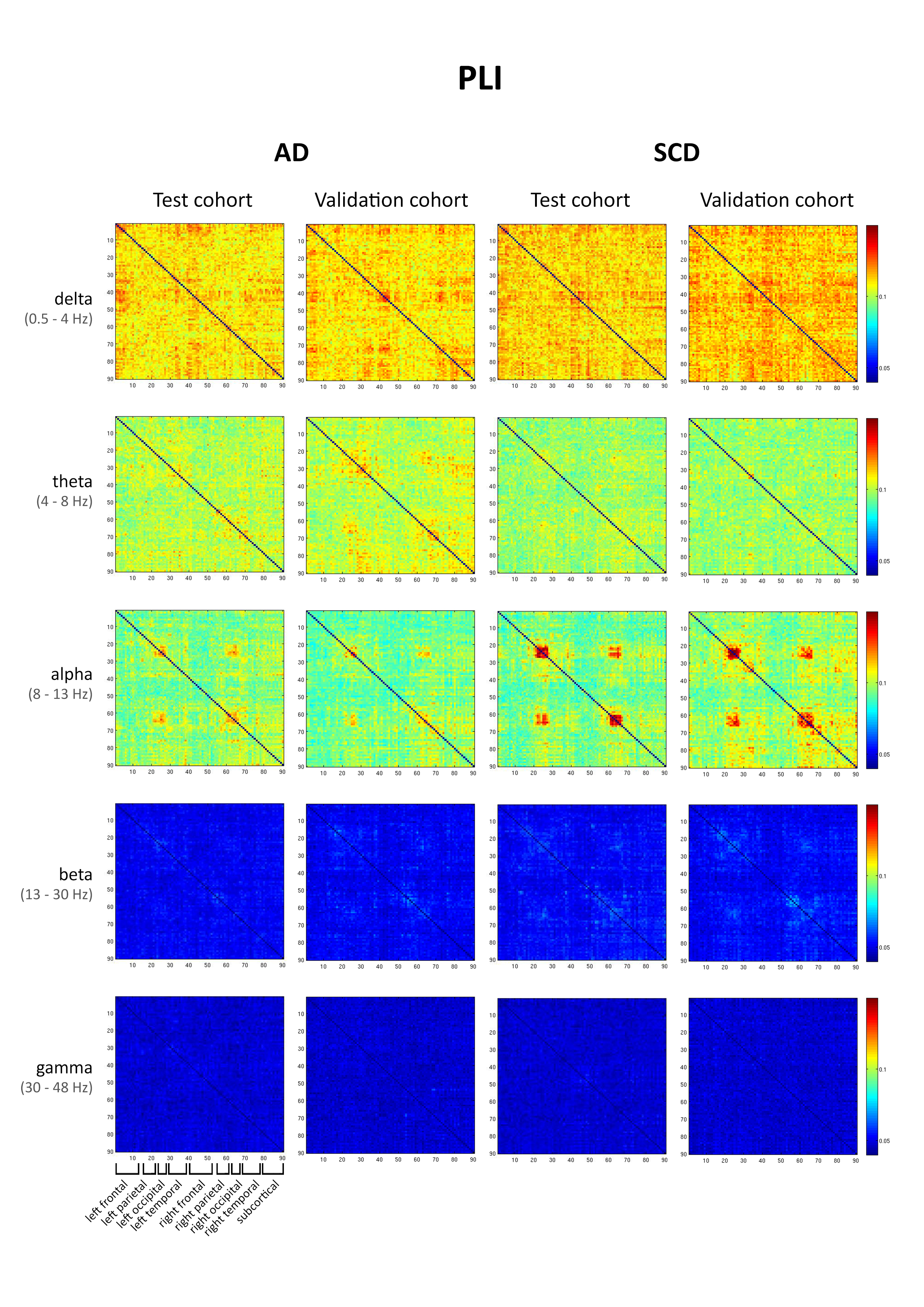

Supplement: Supplementary file 4 — Additional file 4: Fig. S4. Connectivity matrices PLI with same colour scale. Connectivity matrices averaged across all epochs and subjects. Each row represents a different frequency band (delta, theta, alpha, beta and gamma), and each column shows results for the PLI, comparing the test and validation cohort in the AD and SCD groups. All bands show the matrices with the same colour scale. The ROIs are obtained from the AAL atlas, ordered from left to right hemisphere, see Figure 4 and Table S1 for more detailed description. [file 13195_2022_970_MOESM4_ESM.jpg]
