# Supplementary material for: Sensitive and reproducible MEG resting-state metrics of functional connectivity in Alzheimer’s disease
Source: Alzheimers Res Ther. 2022 Feb 26;14:38. doi: 10.1186/s13195-022-00970-4 (PMC8881826; doi:10.1186/s13195-022-00970-4)
Supplement: Supplementary file 5 — Additional file 5: Table S1. AAL atlas regions [44]. Table S2. Global analysis excluding control subjects without biomarkers. Difference in functional connectivity (FC) between SC and AD subjects as estimated by GLM, for the test and validation cohort, excluding subjects with missing biomarkers. Shown are the mean FC values with standard deviations, and the effect sizes as represented by the standardized beta. The AEC-c in the gamma band was analysed using ANOVA on ranks. *bold print indicates a significant (p <0.05) group difference. Print in italics and underscored represents a deviation from the results that were obtained for the analysis that included all subjects. AD: Alzheimer’s disease; SCD: Subjective cognitive decline; GLM: general linear model; AEC-c: corrected amplitude envelope correlation; PLI: phase lag index; SD: standard deviation. Table S3. Global analysis over 5 split-sample iterations + original sample. Difference in functional connectivity (FC) between SCD and AD subjects as estimated by GLM, for the test and validation cohort, for all iterations (five additional samples + original sample). Shown are the mean FC values with standard deviations, and the effect sizes as represented by the standardized beta. The AEC-c in the gamma band was analysed using ANOVA on ranks. *bold print indicates a significant (p <0.05) group difference. AD: Alzheimer’s disease; SCD: Subjective cognitive decline; GLM: general linear model; AEC-c: corrected amplitude envelope correlation; PLI: phase lag index; SD: standard deviation. ICC: Intra-class correlation. Table S4. FDR-corrected Mann-Whitney U outcomes for AEC-c beta band. Depicted are the regional Mann-Whitney U significance outcomes for the AEC-c in the beta band for both the test and validation cohort. The left column depicts the ROI number, while the subsequent columns depict the test outcomes. *signifies p<0.05, corrected. [file 13195_2022_970_MOESM5_ESM.docx]

## **Table S1 - AAL atlas regions (44)**

| **1** | Gyrus Rectus (Left) | **46** | Superior frontal gyrus, dorsolateral  (Right) |
| --- | --- | --- | --- |
| **2** | Olfactory Cortex (Left) | **47** | Middle frontal gyrus (Right) |
| **3** | Superior frontal gyrus, orbital part (Left) | **48** | Inferior frontal gyrus, opercular part (Right) |
| **4** | Superior frontal gyrus, medial orbital  (Left) | **49** | Inferior frontal gyrus, triangular part  (Right) |
| **5** | Middle frontal gyrus, orbital part (Left) | **50** | Superior frontal gyrus, medial (Right) |
| **6** | Inferior frontal gyrus, orbital part (Left) | **51** | Supplementary motor area (Right) |
| **7** | Superior frontal gyrus, dorsolateral  (Left) | **52** | Paracentral lobule (Right) |
| **8** | Middle frontal gyrus (Left) | **53** | Precentral gyrus (Right) |
| **9** | Inferior frontal gyrus, opercular part (Left) | **54** | Rolandic operculum (Right) |
| **10** | Inferior frontal gyrus, triangular part  (Left) | **55** | Postcentral gyrus (Right) |
| **11** | Superior frontal gyrus, medial (Left) | **56** | Superior parietal gyrus (Right) |
| **12** | Supplementary motor area (Left) | **57** | Inferior parietal, but supramarginal and angular gyri (Right) |
| **13** | Paracentral lobule (Left) | **58** | Supramarginal gyrus (Right) |
| **14** | Precentral gyrus (Left) | **59** | Angular gyrus (Right) |
| **15** | Rolandic operculum (Left) | **60** | Precuneus (Right) |
| **16** | Postcentral gyrus (Left) | **61** | Superior occipital gyrus (Right) |
| **17** | Superior parietal gyrus (Left) | **62** | Middle occipital gyrus (Right) |
| **18** | Inferior parietal, but supramarginal and angular gyri (Left) | **63** | Inferior occipital gyrus (Right) |
| **19** | Supramarginal gyrus (Left) | **64** | Calcarine fissure and surrounding cortex (Right) |
| **20** | Angular gyrus (Left) | **65** | Cuneus (Right) |
| **21** | Precuneus (Left) | **66** | Lingual gyrus (Right) |
| **22** | Superior occipital gyrus (Left) | **67** | Fusiform gyrus (Right) |
| **23** | Middle occipital gyrus (Left) | **68** | Heschl gyrus (Right) |
| **24** | Inferior occipital gyrus (Left) | **69** | Superior temporal gyrus (Right) |
| **25** | Calcarine fissure and surrounding cortex (Left) | **70** | Middle temporal gyrus (Right) |
| **26** | Cuneus (Left) | **71** | Inferior temporal gyrus (Right) |
| **27** | Lingual gyrus (Left) | **72** | Temporal pole: superior temporal gyrus (Right) |
| **28** | Fusiform gyrus (Left) | **73** | Temporal pole: middle temporal gyrus (Right) |
| **29** | Heschl gyrus (Left) | **74** | Parahippocampal gyrus (Right) |
| **30** | Superior temporal gyrus (Left) | **75** | Anterior cingulate and paracingulate gyri (Right) |
| **31** | Middle temporal gyrus (Left) | **76** | Median cingulate and paracingulate gyri (Right) |
| **32** | Inferior temporal gyrus (Left) | **77** | Posterior cingulate gyrus (Right) |
| **33** | Temporal pole: superior temporal gyrus (Left) | **78** | Insula (Right) |
| **34** | Temporal pole: middle temporal gyrus (Left) | **79** | Hippocampus (Left) |
| **35** | Parahippocampal gyrus (Left) | **80** | Hippocampus (Right) |
| **36** | Anterior cingulate and paracingulate gyri (Left) | **81** | Amygdala (Left) |
| **37** | Median cingulate and paracingulate gyri (Left) | **82** | Amygdala (Right) |
| **38** | Posterior cingulate gyrus (Left) | **83** | Caudate (Left) |
| **39** | Insula (Left) | **84** | Caudate (Right) |
| **40** | Gyrus Rectus (Right) | **85** | Putamen (Left) |
| **41** | Olfactory Cortex (Right) | **86** | Putamen (Right) |
| **42** | Superior frontal gyrus, orbital part (Right) | **87** | Pallidum (Left) |
| **43** | Superior frontal gyrus, medial orbital  (Right) | **88** | Pallidum (Right) |
| **44** | Middle frontal gyrus, orbital part (Right) | **89** | Thalamus (Left) |
| **45** | Inferior frontal gyrus, orbital part (Right) | **90** | Thalamus (Right) |

***Table S2 – Global analysis excluding control subjects without biomarkers***

|  | **Test cohort n=54** | | | | | **Validation cohort n=49** | | | |
| --- | --- | --- | --- | --- | --- | --- | --- | --- | --- |
|  | **AD (n=28)** | **SCD (n=26)** | **GLM** | | **AD (n=29)** | | **SCD (n=20)** | **GLM** | |
| **AEC-c (global)** | mean ± SD | mean ± SD | standardised beta (β) | *p*-value | mean ± SD | | mean ± SD | standardised beta (β) | *p*-value |
| Delta (0.5 - 4 Hz) | **0.517 ± 0.013** | **0.509 ± 0.007** | **0.342** | **0.011*** | ***0.514 ± 0.012*** | | ***0.508 ± 0.006*** | ***0.285*** | ***0.047**** |
| Theta (4- 8 Hz) | 0.520  **±** 0.012 | 0.517 ± 0.013 | 0.131 | 0.345 | 0.520 ± 0.013 | | 0.515 ± 0.011 | 0.195 | 0.180 |
| Alpha (8 – 13 Hz) | *0.521 ± 0.013* | *0.528 ± 0.014* | *-0.253* | *0.065* | *0.520 ± 0.012* | | *0.527 ± 0.014* | *-0.258* | *0.073* |
| Beta (13 – 30 Hz) | **0.514 ± 0.006** | **0.521 ± 0.009** | **-0.407** | **0.002*** | **0.514 ± 0.007** | | **0.519 ± 0.007** | **-0.364** | **0.010*** |
| Gamma (30 – 48 Hz)∞ | 0.504  **±** 0.007 | 0.502  **±** 0.002 | 0.217 | 0.115 | 0.502 **±** 0.004 | | 0.502 **±** 0.003 | -0.088 | 0.546 |
| **PLI (global)** |  |  |  |  |  | |  |  |  |
| Delta (0.5 - 4 Hz) | **0.111 ± 0.004** | **0.113 ± 0.004** | **-0.298** | **0.029*** | **0.111 ± 0.005** | | **0.114 ± 0.003** | **-0.401** | **0.004*** |
| Theta (4 - 8 Hz) | **0.101 ± 0.005** | **0.098 ± 0.004** | **0.350** | **0.009*** | **0.103 ± 0.006** | | **0.098 ± 0.003** | **0.404** | **0.004*** |
| Alpha (8 – 13 Hz) | 0.097 ± 0.008 | 0.096 ± 0.006 | 0.071 | 0.612 | **0.094 ± 0.007** | | **0.101 ± 0.009** | **-0.392** | **0.005*** |
| Beta (13 – 30 Hz) | **0.052 ± 0.002** | **0.053 ± 0.003** | **-0.279** | **0.041*** | ***0.052 ± 0.002*** | | ***0.053 ± 0.003*** | ***-0.289*** | ***0.044**** |
| Gamma (3 – 48 Hz) | 0.047  **±** 0.002 | 0.047 ± 0.002 | 0.191 | 0.166 | 0.047 ± 0.001 | | 0.047 ± 0.001 | --0.021 | 0.887 |

*Difference in functional connectivity (FC) between SC and AD subjects as estimated by GLM, for the test and validation cohort, excluding subjects with missing biomarkers. Shown are the mean FC values with standard deviations, and the effect sizes as represented by the standardized beta. The AEC-c in the gamma band was analysed using ANOVA on ranks. *bold print indicates a significant (p <0.05) group difference. Print in italics and underscored represents a deviation from the results that were obtained for the analysis that included all subjects. AD: Alzheimer’s disease; SCD: Subjective cognitive decline; GLM: general linear model; AEC-c: corrected amplitude envelope correlation; PLI: phase lag index; SD: standard deviation.*

***Table S3 - Global analysis over 5 split-sample iterations + original sample***

|  | **Test cohort (n=53-59)** | | | **Validation cohort (n=54-60)** | | |
| --- | --- | --- | --- | --- | --- | --- |
|  | **AD (n=26-31)** | **HC (n=24-31)** | **GLM** | **AD (n=26-31)** | **HC (n=24-32)** | **GLM** |
| **AEC-c (global)** | mean ± SD | mean ± SD | standardised beta (β) | mean ± SD | mean ± SD | standardised beta (β) |
| Delta  (0.5 - 4 Hz) | 0.515 ± 0.013 | 0.509 ± 0.008 | 0.276 | 0.516 ± 0.013 | 0.509 ± 0.007 | 0.325 |
| Theta  (4- 8 Hz) | 0.520 ± 0.013 | 0.518 ± 0.013 | 0.098 | 0.520 ± 0.013 | 0.518 ± 0.013 | 0.073 |
| Alpha  (8 – 13 Hz) | 0.520 ± 0.013 | 0.529 ± 0.016 | -0.292 | 0.521 ± 0.013 | 0.531 ± 0.018 | -0.321 |
| Beta  (13 – 30 Hz) | 0.514 ± 0.007 | 0.523 ± 0.014 | -0.366 | 0.515 ± 0.006 | 0.523 ± 0.011 | -0.458 |
| Gamma  (30 – 48 Hz) | 0.503 ± 0.007 | 0.502 ± 0.003 | 0.101 | 0.503 ± 0.005 | 0.502 ± 0.004 | 0.021 |
| **PLI (global)** |  |  |  |  |  |  |
| Delta  (0.5 - 4 Hz) | 0.111 ± 0.004 | 0.113 ± 0.004 | -0.248 | 0.111 ± 0.005 | 0.114 ± 0.004 | -0.342 |
| Theta  (4 - 8 Hz) | 0.102 ± 0.006 | 0.097 ± 0.004 | 0.443 | 0.101 ± 0.005 | 0.098 ± 0.004 | 0.347 |
| Alpha  (8 – 13 Hz) | 0.096 ± 0.008 | 0.097 ± 0.008 | -0.131 | 0.096 ± 0.007 | 0.098 ± 0.008 | -0.159 |
| Beta  (13 – 30 Hz) | 0.052 ± 0.002 | 0.053 ± 0.003 | -0.239 | 0.052 ± 0.002 | 0.053 ± 0.003 | -0.306 |
| Gamma  (3 – 48 Hz) | 0.048 ± 0.002 | 0.048 ± 0.002 | 0.065 | 0.048 ± 0.002 | 0.048 ± 0.002 | -0.010 |

| **Reproducibility across iterations** | **0x** | **1x** | **2x** | **3x** | **4x** | **5x** | **6x** |
| --- | --- | --- | --- | --- | --- | --- | --- |

*Difference in functional connectivity (FC) between SCD and AD subjects as estimated by GLM, for the test and validation cohort****,*** *for all iterations (five additional samples + original sample). Shown are the mean FC values with standard deviations, and the effect sizes as represented by the standardized beta. The AEC-c in the gamma band was analysed using ANOVA on ranks. *bold print indicates a significant (p <0.05) group difference. AD: Alzheimer’s disease; SCD: Subjective cognitive decline; GLM: general linear model; AEC-c: corrected amplitude envelope correlation; PLI: phase lag index; SD: standard deviation. ICC: Intra-class correlation*

***Table S4– FDR-corrected Mann-Whitney U outcomes for AEC-c beta band***

| ROI | AAL atlas | Test cohort | Validation cohort |
| --- | --- | --- | --- |
| 1 | Gyrus Rectus (Left) | 0,048* | 0,028 |
| 2 | Olfactory Cortex (Left) | 0,031* | 0,017* |
| 3 | Superior frontal gyrus, orbital part (Left) | 0,065 | 0,032* |
| 4 | Superior frontal gyrus, medial orbital  (Left) | 0,015* | 0,100 |
| 5 | Middle frontal gyrus, orbital part (Left) | 0,041* | 0,019* |
| 6 | Inferior frontal gyrus, orbital part (Left) | 0,103 | 0,016* |
| 7 | Superior frontal gyrus, dorsolateral  (Left) | 0,047* | 0,032* |
| 8 | Middle frontal gyrus (Left) | 0,022* | 0,031* |
| 9 | Inferior frontal gyrus, opercular part (Left) | 0,011* | 0,019* |
| 10 | Inferior frontal gyrus, triangular part  (Left) | 0,020* | 0,013* |
| 11 | Superior frontal gyrus, medial (Left) | 0,060 | 0,081 |
| 12 | Supplementary motor area (Left) | 0,013* | 0,012* |
| 13 | Paracentral lobule (Left) | 0,032* | 0,037* |
| 14 | Precentral gyrus (Left) | 0,035* | 0,053 |
| 15 | Rolandic operculum (Left) | 0,011* | 0,003* |
| 16 | Postcentral gyrus (Left) | 0,007* | 0,048* |
| 17 | Superior parietal gyrus (Left) | <0,001* | <0,001* |
| 18 | Inferior parietal, but supramarginal and angular gyri (Left) | <0,001* | <0,001* |
| 19 | Supramarginal gyrus (Left) | <0,001* | 0,008* |
| 20 | Angular gyrus (Left) | <0,001* | <0,001* |
| 21 | Precuneus (Left) | <0,001* | 0,006* |
| 22 | Superior occipital gyrus (Left) | <0,001* | <0,001* |
| 23 | Middle occipital gyrus (Left) | <0,001* | <0,001* |
| 24 | Inferior occipital gyrus (Left) | 0,009* | <0,001* |
| 25 | Calcarine fissure and surrounding cortex (Left) | 0,005* | 0,003* |
| 26 | Cuneus (Left) | <0,001* | <0,001* |
| 27 | Lingual gyrus (Left) | 0,003* | 0,003* |
| 28 | Fusiform gyrus (Left) | 0,003* | <0,001* |
| 29 | Heschl gyrus (Left) | 0,010* | 0,003* |
| 30 | Superior temporal gyrus (Left) | 0,017* | 0,003* |
| 31 | Middle temporal gyrus (Left) | 0,003* | <0,001* |
| 32 | Inferior temporal gyrus (Left) | <0,001* | <0,001* |
| 33 | Temporal pole: superior temporal gyrus (Left) | 0,071 | 0,013* |
| 34 | Temporal pole: middle temporal gyrus (Left) | 0,029* | 0,038* |
| 35 | Parahippocampal gyrus (Left) | 0,015* | <0,001* |
| 36 | Anterior cingulate and paracingulate gyri (Left) | 0,028* | 0,016* |
| 37 | Median cingulate and paracingulate gyri (Left) | 0,007* | 0,012* |
| 38 | Posterior cingulate gyrus (Left) | <0,001* | <0,001* |
| 39 | Insula (Left) | 0,033* | 0,003* |
| 40 | Gyrus Rectus (Right) | 0,009* | 0,030* |
| 41 | Olfactory Cortex (Right) | 0,007* | 0,020* |
| 42 | Superior frontal gyrus, orbital part (Right) | 0,011* | 0,038* |
| 43 | Superior frontal gyrus, medial orbital  (Right) | 0,011* | 0,093 |
| 44 | Middle frontal gyrus, orbital part (Right) | 0,011* | 0,033* |
| 45 | Inferior frontal gyrus, orbital part (Right) | 0,020* | 0,030* |
| 46 | Superior frontal gyrus, dorsolateral  (Right) | 0,011* | 0,048* |
| 47 | Middle frontal gyrus (Right) | 0,033* | 0,029* |
| 48 | Inferior frontal gyrus, opercular part (Right) | 0,011* | 0,008* |
| 49 | Inferior frontal gyrus, triangular part  (Right) | 0,020* | 0,104 |
| 50 | Superior frontal gyrus, medial (Right) | 0,016* | 0,038* |
| 51 | Supplementary motor area (Right) | 0,068 | 0,066 |
| 52 | Paracentral lobule (Right) | 0,011* | 0,032* |
| 53 | Precentral gyrus (Right) | 0,011* | 0,029* |
| 54 | Rolandic operculum (Right) | 0,011* | 0,003* |
| 55 | Postcentral gyrus (Right) | 0,010* | 0,029* |
| 56 | Superior parietal gyrus (Right) | <0,001* | 0,006* |
| 57 | Inferior parietal, but supramarginal and angular gyri (Right) | <0,001* | <0,001* |
| 58 | Supramarginal gyrus (Right) | <0,001* | 0,011* |
| 59 | Angular gyrus (Right) | <0,001* | <0,001* |
| 60 | Precuneus (Right) | <0,001* | 0,006* |
| 61 | Superior occipital gyrus (Right) | <0,001* | <0,001* |
| 62 | Middle occipital gyrus (Right) | <0,001* | <0,001* |
| 63 | Inferior occipital gyrus (Right) | <0,001* | <0,001* |
| 64 | Calcarine fissure and surrounding cortex (Right) | <0,001* | <0,001* |
| 65 | Cuneus (Right) | <0,001* | 0,003* |
| 66 | Lingual gyrus (Right) | <0,001* | 0,003* |
| 67 | Fusiform gyrus (Right) | <0,001* | <0,001* |
| 68 | Heschl gyrus (Right) | 0,003* | 0,011* |
| 69 | Superior temporal gyrus (Right) | <0,001* | 0,005* |
| 70 | Middle temporal gyrus (Right) | <0,001* | <0,001* |
| 71 | Inferior temporal gyrus (Right) | <0,001* | <0,001* |
| 72 | Temporal pole: superior temporal gyrus (Right) | 0,055 | 0,025* |
| 73 | Temporal pole: middle temporal gyrus (Right) | 0,015* | 0,069 |
| 74 | Parahippocampal gyrus (Right) | 0,010* | 0,003* |
| 75 | Anterior cingulate and paracingulate gyri (Right) | 0,020* | 0,072 |
| 76 | Median cingulate and paracingulate gyri (Right) | 0,007* | 0,010* |
| 77 | Posterior cingulate gyrus (Right) | <0,001* | <0,001* |
| 78 | Insula (Right) | 0,076 | 0,005* |
| 79 | Hippocampus (Left) | 0,005* | 0,003* |
| 80 | Hippocampus (Right) | 0,003* | 0,005* |
| 81 | Amygdala (Left) | 0,016* | 0,003* |
| 82 | Amygdala (Right) | 0,028* | 0,021* |
| 83 | Caudate (Left) | 0,022* | 0,011* |
| 84 | Caudate (Right) | 0,011* | 0,030* |
| 85 | Putamen (Left) | 0,020* | 0,006* |
| 86 | Putamen (Right) | 0,033* | 0,005* |
| 87 | Pallidum (Left) | 0,013* | 0,003* |
| 88 | Pallidum (Right) | 0,028* | 0,017* |
| 89 | Thalamus (Left) | 0,007* | 0,012* |
| 90 | Thalamus (Right) | 0,003* | 0,003* |

*Depicted are the regional Mann-Whitney U significance outcomes for the AEC-c in the beta band for both the test and validation cohort. The left column depicts the ROI number, while the subsequent columns depict the test outcomes. *signifies p<0.05, corrected.*
